# Assessment of linkage disequilibrium patterns between structural variants and single nucleotide polymorphisms in three commercial chicken populations

**DOI:** 10.1186/s12864-022-08418-7

**Published:** 2022-03-09

**Authors:** Johannes Geibel, Nora Paulina Praefke, Steffen Weigend, Henner Simianer, Christian Reimer

**Affiliations:** 1 Animal Breeding and Genetics Group, Department of Animal Sciences, University of Goettingen, Göttingen, Germany; 2 Center for Integrated Breeding Research, University of Goettingen, Göttingen, Germany; 3 Institute of Farm Animal Genetics, Friedrich-Loeffler-Institut, Neustadt, Germany

**Keywords:** Chickens, Single nucleotide polymorphisms, Structural variants, Linkage disequilibrium

## Abstract

**Background:**

Structural variants (SV) are causative for some prominent phenotypic traits of livestock as different comb types in chickens or color patterns in pigs. Their effects on production traits are also increasingly studied. Nevertheless, accurately calling SV remains challenging. It is therefore of interest, whether close-by single nucleotide polymorphisms (SNPs) are in strong linkage disequilibrium (LD) with SVs and can serve as markers. Literature comes to different conclusions on whether SVs are in LD to SNPs on the same level as SNPs to other SNPs. The present study aimed to generate a precise SV callset from whole-genome short-read sequencing (WGS) data for three commercial chicken populations and to evaluate LD patterns between the called SVs and surrounding SNPs. It is thereby the first study that assessed LD between SVs and SNPs in chickens.

**Results:**

The final callset consisted of 12,294,329 bivariate SNPs, 4,301 deletions (DEL), 224 duplications (DUP), 218 inversions (INV) and 117 translocation breakpoints (BND). While average LD between DELs and SNPs was at the same level as between SNPs and SNPs, LD between other SVs and SNPs was strongly reduced (DUP: 40%, INV: 27%, BND: 19% of between-SNP LD). A main factor for the reduced LD was the presence of local minor allele frequency differences, which accounted for 50% of the difference between SNP – SNP and DUP – SNP LD. This was potentially accompanied by lower genotyping accuracies for DUP, INV and BND compared with SNPs and DELs. An evaluation of the presence of tag SNPs (SNP in highest LD to the variant of interest) further revealed DELs to be slightly less tagged by WGS SNPs than WGS SNPs by other SNPs. This difference, however, was no longer present when reducing the pool of potential tag SNPs to SNPs located on four different chicken genotyping arrays.

**Conclusions:**

The results implied that genomic variance due to DELs in the chicken populations studied can be captured by different SNP marker sets as good as variance from WGS SNPs, whereas separate SV calling might be advisable for DUP, INV, and BND effects.

**Supplementary Information:**

The online version contains supplementary material available at 10.1186/s12864-022-08418-7.

## Background

A type of genomic variation that affects large regions of the genome is caused by structural variants (SV). SVs can alter the total genome size by deleting (deletions, DEL), duplicating (duplications, DUP) or inserting (insertions, INS) longer stretches of DNA (unbalanced SV). Those SVs are often referred to as copy number variations (CNV). In contrast, inversions (INV) and translocations (TRA) do not affect the length of the genome (balanced SV) [1]. Especially unbalanced SVs are assumed to come with a strong functional impact on the phenotype, e.g. by strong deleterious effects of DELs which can remove complete genes [2] or by DUPs that increase numbers of cis-regulatory elements [2, 3]. SVs and complex combinations of multiple SVs are also known to be causative for some of the most prominent phenotypic breed characteristics of livestock breeds as walnut- and rose comb in chickens [4] or belted color patterns and dominant-white color in pigs [5].

The power for detection of SVs of certain types and sizes, however, is highly technology-dependent in various aspects [1]. During the last two decades, technologies evolved that increased the resolution and accuracy of SV detection at the submicroscopic level. Array-based comparative genomic hybridization (aCGH) allowed the detection of long CNVs > 35 kb [2]. The development and increased use of microarrays led to technologies that either detect DELs from characteristics of population-level single nucleotide polymorphism (SNP) genotypes [6, 7] or utilized signal intensity information [8]. The increasing availability of short-read sequences during the last decade led to the development of multiple SV detection algorithms which use read depth distributions [1, 9] and/ or information from split reads and insert size distributions of paired-end reads, potentially combined with local assembly procedures [1, 10–12]. However, short-read-based methods still come with a variety of limitations due to the short read sizes which highly vary between the algorithms [1, 13] and especially a general deficit in calling INS [14]. Therefore, current state-of-the-art methods nowadays utilize the information of PacBio or Nanopore long-read sequencing or linked-read technologies as HI-C [15], but the availability of these types of sequencing data is still very limited for the majority of intensively researched livestock species.

Other than for SVs, the use of SNPs has become routine over the last two decades. Therefore, large whole-genome-sequencing (WGS) reference panels [16, 17] and collections of individuals, which were genotyped by microarrays and phenotyped in routine breeding programs or during large-scale research projects [18], exist. Given the complexity of SV detection, it is of interest to know which part of the effects of SVs on the phenotype is already captured by potential linkage disequilibrium (LD) between the SV of interest and nearby SNPs. Strong LD would allow for the inclusion of those effects in e.g. genomic prediction without the need for a separate SV analysis.

LD between two variants can be measured using a variety of estimators (reviewed e.g. by Qanbari [19]), of which the squared correlation of haplotypes (${\mathrm{r}}^{2}$) is probably the most prominent one. It can be interpreted as the amount of information of a variant that is captured by another one. However, its upper limit is defined by the difference in minor allele frequency (ΔMAF) between the two variants [20]. The overall strength of LD is highly population depended and closely linked to the effective population size [19]. LD thereby shows a characteristic decay pattern of mean LD by distance. However, for many applications as genome-wide association studies (GWAS), the interest is more in the maximum observed LD of a causal variant to a close-by so-called tag SNP, which can capture the effect as a marker genotype.

By now, a bunch of studies has addressed the question of LD between SVs and surrounding SNPs in humans with contrasting results. Generally, common DELs were shown to be in good LD to SNPs by most of the studies [6, 21–24], but some found this LD to be weaker than SNP – SNP LD [25, 26]. Literature additionally suggests, that rare DELs are weaker tagged (tag SNP is SNP with highest LD to the variant within a defined distance) than common DELs [22, 27] and DUP were in weaker LD to SNPs than DELs [22, 26, 28]. It was additionally shown that the availability of tag SNPs for SVs depends on the SNP panel used (WGS vs. different arrays) [22–24]. A further effect that was found is the location of the SV on the genome. Regions of segmental duplications are known to trigger recurrent SV formation by non-allelic homologous recombination and therefore lead to SV hotspots [1, 29]. A closer look at those regions by Locke et al*.* [30] found very few of those CNV to be tagged by surrounding SNPs.

Reduced LD between SNPs and SVs can have diverse reasons. A main factor is the increased possibility of the occurrence of recurrent mutations in regions of low sequence complexity by non-allelic homologous recombination (NAHR) [29]. SVs from recurrent mutational events then show reduced LD to variants from a unique mutational event [6, 30]. LD between SNPs and SVs may further be decreased by different selectional properties of SNPs and SV [31], MAF differences between SVs and SNPs [20], or ascertainment of SNPs for arrays that excludes regions of high structural complexity due to technical reasons [32]. Additionally, known problems with SV calling accuracy [1] may lead to a high share of false-positive SV calls and therefore on average low LD to more accurately called SNPs.

For livestock, results on SV – SNP LD are very rare, even though a high number of publications targeted SV. Based on a GWAS on 26,362 Holstein dairy cattle 50 k genotypes, Xu et al*.* [33] found a quarter of CNVs that were significantly associated with milk traits not being tagged by adjacent SNPs. The same was observed by Lee et al*.* [32] who investigated functional and population genetic features of CNV regions in two dairy cattle breeds, also called from a 50 k SNP array. They identified a weak linkage between CNV regions and SNPs, which was slightly stronger between DELs and SNPs than between DUPs and SNPs. Wang et al*.* [34] included a local LD analysis around CNVs (called from SNP arrays) that were significantly associated with production traits in pigs. Four out of eight significantly associated CNVs overlapped haploblocks of non-significant SNPs, but only one CNV was found 300 kb downstream of significantly associated SNPs. Note that this, however, may also have been an artifact of a much stronger correction for multiple testing in SNPs than in CNVs.

In chickens, a variety of studies investigated CNVs on a quantitative basis. The studies either used aCGH [35–39], utilized signal information of SNP arrays [40–45] via PennCNV [8], or read depth information of short-read sequences [46–50]. There were only three studies that also included non-CNV SVs [46, 50, 51]. None of the studies analyzed the LD patterns of the variants.

### Aim of the study

This is the first study that assessed SV – SNP LD in chickens to investigate the usefulness of SNP markers in capturing SV-based genomic variance. We, therefore, identified SVs from paired-end short-read sequences in three commercial chicken populations (white layers, brown layers, broilers), thoroughly described the SV callset, and assessed the strength of LD between those SVs and SNPs. We also identified major reasons for some existing differences to SNP – SNP LD and evaluated the performance of four available SNP arrays to tag SVs.

## Results

### Calling results and description of variants

For the study, paired-end short-read sequences of 90 chickens from three populations (25 commercial white layers, WL; 25 commercial brown layers, BL; 40 commercial broiler chickens, BR) were used. The raw data was first published by Qanbari et al*.* [52] who described the studied populations in more detail. SNP genotypes were retrieved from a previous study [53]. SVs were called by a consensus calling approach, which used three paired-end and split-read-based tools, followed by a strict filtering procedure that further utilized read-depth and SNP information. Finally, the remaining SV calls were visually checked by evaluating samplots [54] for each variant, the merged SNP and SV set was phased, and missing genotypes were imputed. The filtering procedure retained 12,294,329 bivariate SNPs, 4,301 DELs, 224 DUPs, 218 INVs, and 117 translocation breakpoints (break ends; BND) on chromosomes 1—33. Note that all INS were filtered out due to missing support by at least two variant callers.

**Fig. 1:**
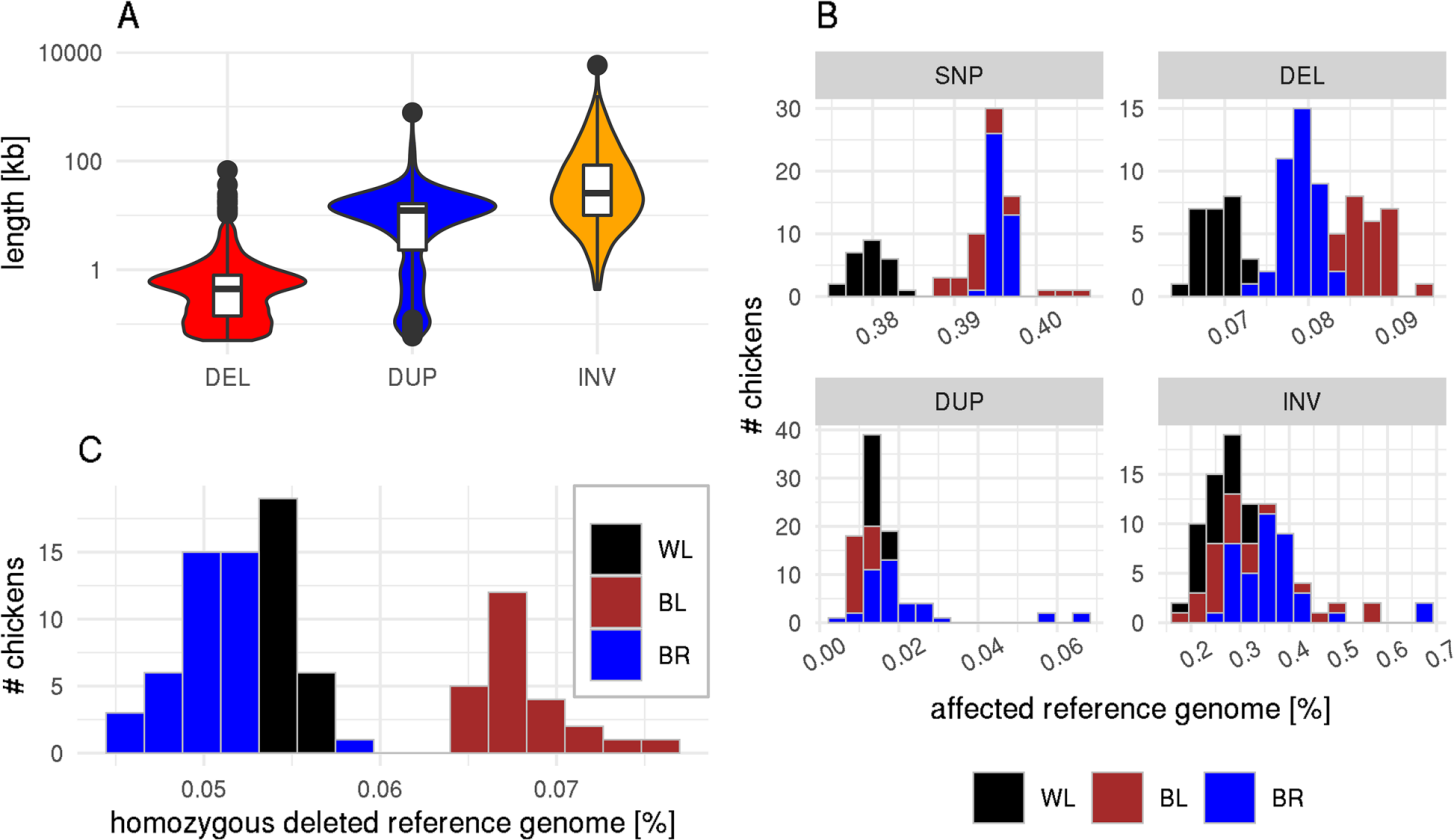
Length distribution of SVs (**A**), percent of affected autosomal reference genome by individual and variation type (**B**), and percent of homozygously deleted reference genome by individuals (**C**). The size in B is calculated as the average between the haplotypes of an individual affected by the non-reference allele. Note the log-scaled y-axis in A. Per-breed bars in the histograms are stacked on each other

Figure 1 A shows the length distribution of the called SVs. DELs were on average shortest with a median of 443 bp and a maximum of 67,037 bp. DUPs (median = 12,285 bp; maximum = 778,041 bp) were larger than DELs and INVs were largest (median = 25,643 bp; maximum = 5,795,187 bp). BNDs only indicate translocation breakpoints and, therefore, do not come with length information. The called SNPs in total accounted for 1.28% of the autosomal reference genome length, while DELs covered 0.35%, DUPs 0.39%, and INVs 2.80% of the chicken genome. The distributions by individuals can be found in Fig. 1B. We additionally checked how much of the autosomal reference genome is homozygously deleted in the chickens. This number varied from 0.045% (135 kb) to 0.076% (727 kb) with BL showing a larger size of homozygously deleted reference genome than WL and BR (Fig. 1C)

We further checked for chromosome-wise differences in the number of called variants by regressing the relative number of called variants per chromosome on the relative chromosome length (Fig. S3). SNPs did not show any difference to the line of identity (slope = 1.00, *p* = 1.00), while DELs (slope = 1.28, *p* = 1.4e-4) and INVs (slope = 1.39, *p* = 6.1e-9) showed a significant bias towards larger chromosomes. DUPs (slope = 1.13, *p* = 0.34) and BNDs (slope = 1.14, *p* = 0.17) also showed a numerical bias towards larger chromosomes, which, however, was not significant. Note that the *R*^*2*^ value of the model was comparably small with 0.39.

**Fig. 2:**
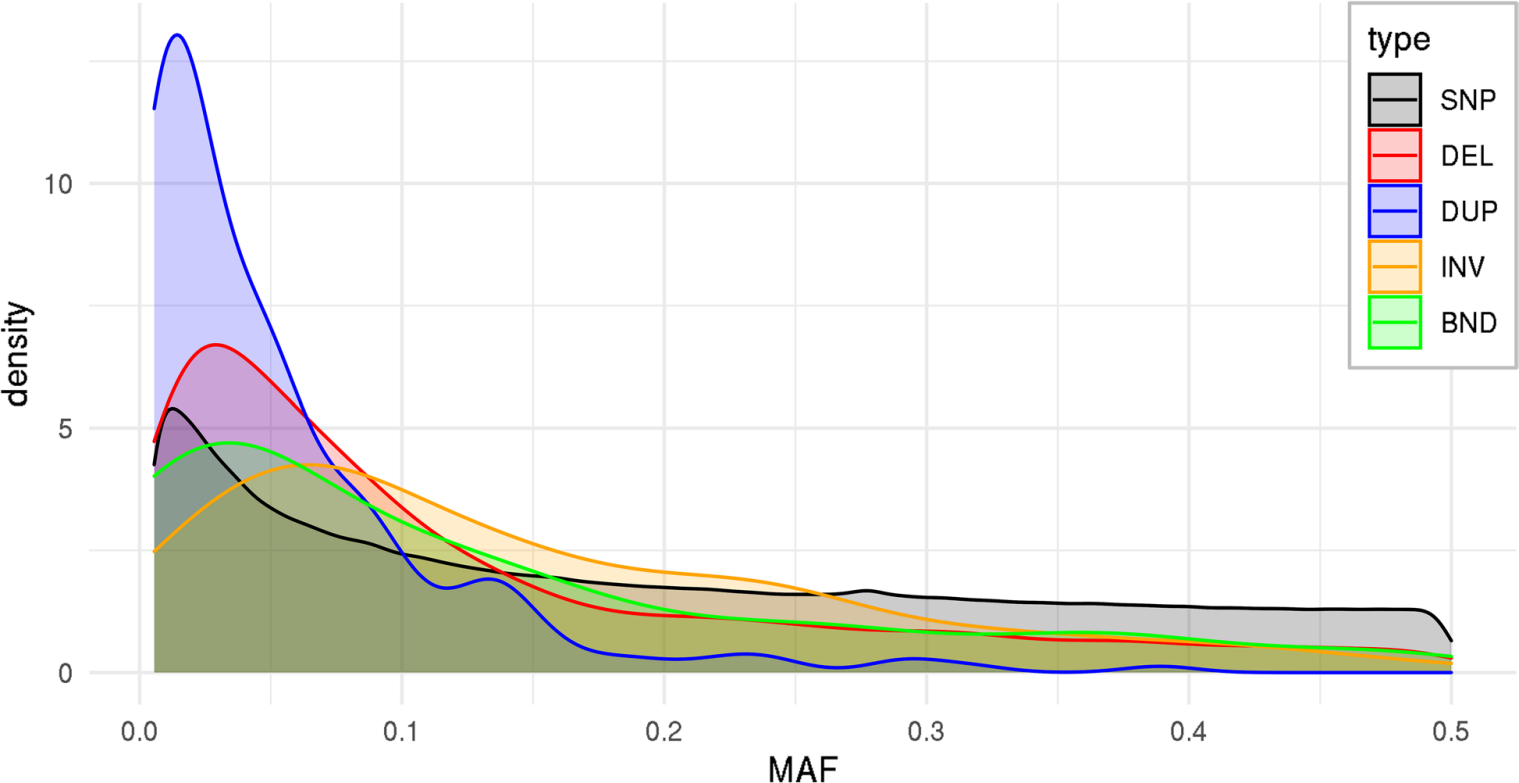
Distribution of minor allele frequency (MAF) across all samples by variant type

Distributions of minor allele frequencies (MAF; Fig. 2) revealed a slight (DEL) to strong (DUP) shift towards rare variants compared with SNPs for DELs and DUPs, while INVs and BNDs showed a slight shift towards more common variants.

Variant effect predictions of Ensembl-vep [55] classified 98.48% of the impacts of SNPs on genes as MODIFIER, 1.14% as LOW, 0.37 as MODERATE and only 0.01% as HIGH. DEL impacts were classified only in 0.41% of the cases other than MODIFIER (MODERATE = 0.01%; HIGH = 0.40%), while DUP impacts were classified as HIGH in 9.95% of the cases (MODIFIER = 90.05%). In contrast, INV and BND impacts were completely classified as MODIFIER. Further results of VEP are summarized in Fig. S4.

### LD decay

To assess the information content of SNPs on SVs, we calculated the LD between SVs and all bivariate SNPs up to 100 kb apart from the breakpoints as squared haplotype correlation (${\mathrm{r}}^{2}$). Note, that SNPs that were located on SVs were excluded from the analysis, as their calls may be directly influenced by the SV. To get a baseline for comparisons, we also calculated the SNP – SNP LD within this distance.

**Fig. 3:**
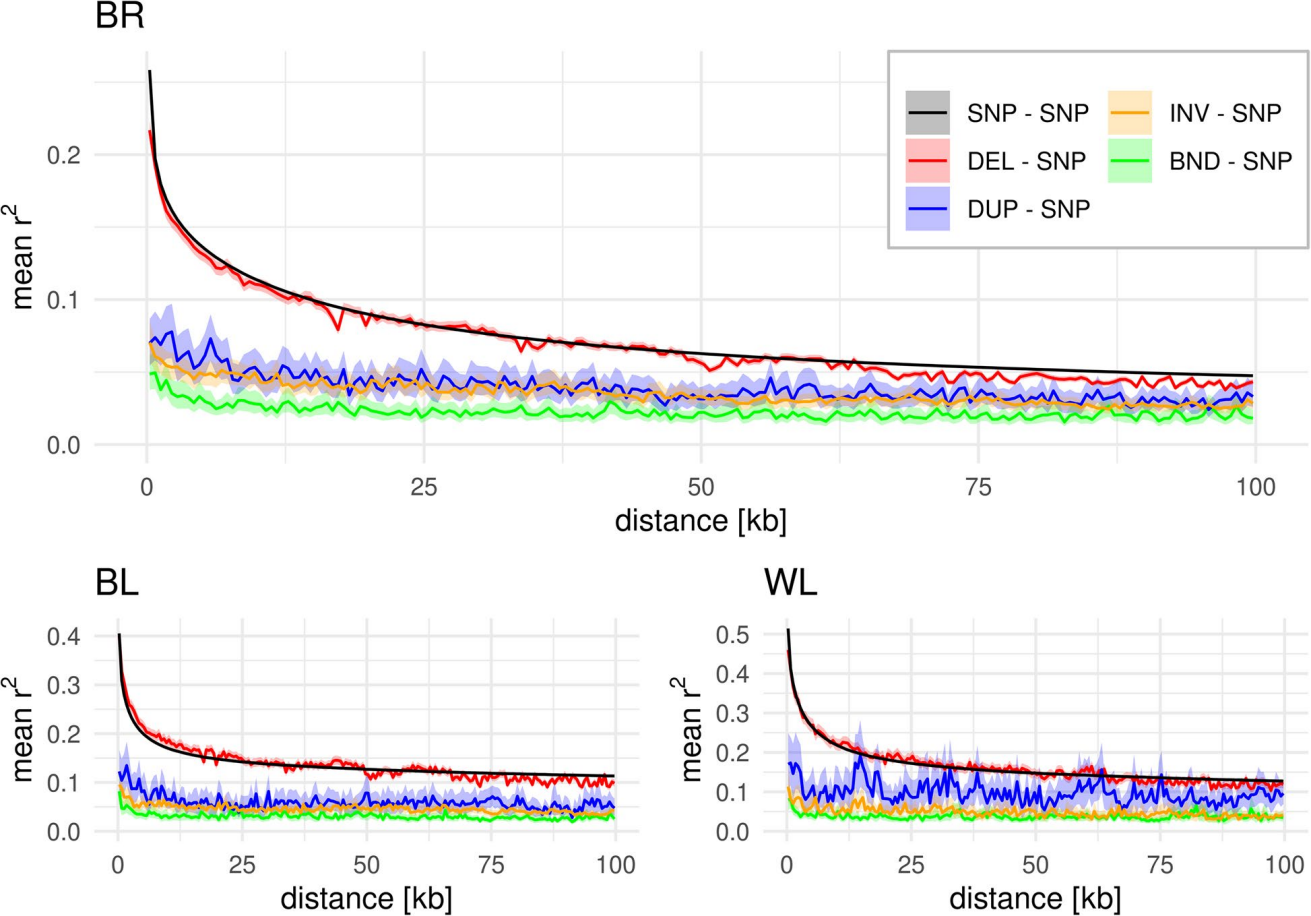
LD decay in the broiler (BR), brown layer (BL) and white layer (WL) chickens. The LD is presented as mean ${r}^{2}$ in 500 bp distance bins and the shaded areas represent Bonferroni-corrected 95% bootstrap confidence intervals. For SNP – SNP distance bins with > 1 M ${r}^{2}$ values, no confidence intervals were estimated

Mean SNP – SNP ${\mathrm{r}}^{2}$ was highest in WL (0.51 within 500 bp), followed by BL (0.41) and BR (0.26). The DEL – SNP LD decay curve follows closely the pattern of the SNP – SNP LD decay (Fig. 3). Even though the level of LD was strongly reduced for the other variant types, a slight decay curve with increasing distance was still noticeable. Due to the small number of called DUPs in WL, the decay curve strongly fluctuated in this population. However, BR and BL gave some evidence that the DUP – SNP and INV – SNP decay curves were comparable, while BND – SNP decay came with a slightly lower level of LD.

To quantify the difference in LD between variants and populations and account for the population-specific level of LD, we expressed the mean LD in the 500 bp bins relative to the SNP – SNP LD and further averaged those values for the first 10 bins (Table 1). This revealed comparable values within variants and across populations of less than 12% difference. Across all populations, DEL – SNP LD was on the same level as SNP – SNP LD, while DUP – SNP LD was ~ 40%, INV – SNP ~ 27% and BND – SNP ~ 19% of SNP – SNP LD within 5 kb distance. Note that the relative ${\mathrm{r}}^{2}$ was not necessarily constant across the complete range of 100 kb (Fig. S5).

**Table 1:** SV – SNP ${r}^{2}$/${r}_{S}^{2}$ relative to the SNP – SNP ${r}^{2}$/${r}_{S}^{2}$

Type	All			BR			BL			WL		
	${r}^{2}$^a^	${r}_{S}^{2}$^b^	Δ^c^	${r}^{2}$^a^	${r}_{S}^{2}$^b^	Δ^c^	${r}^{2}$^a^	${r}_{S}^{2}$^b^	Δ^c^	${r}^{2}$^a^	${r}_{S}^{2}$^a^	Δ^c^
**DEL – SNP**	100.1 _± 6.1_	98.8 _± 4.3_	-1.3	95.4 _± 4.1_	94.2 _± 2.5_	-1.2	107.0 _± 3.2_	103.2 _± 1.2_	-3.8	98.1 _± 3.4_	98.8 _± 2.5_	0.7
**DUP – SNP**	39.9 _± 6.8_	68.2 _± 8.9_	28.3	39.5 _± 5.8_	66.7 _± 4.3_	27.2	41.1 _± 7.0_	65.6 _± 9.9_	24.5	39.1 _± 8.1_	72.3 _± 10.5_	33.2
**INV – SNP**	26.8 _± 5.2_	46.0 _± 4.3_	19.2	32.6 _± 2.4_	46.8 _± 1.7_	14.2	26.0 _± 2.4_	50.1 _± 3.4_	24.1	21.6 _± 3.1_	50.1 _± 6.2_	28.5
**BND – SNP**	18.5 _± 3.6_	46.9 _± 5.4_	28.4	22.4 _± 2.3_	50.4 _± 3.7_	28.0	18.0 _± 1.9_	44.6 _± 5.1_	26.6	15.3 _± 2.0_	45.5 _± 5.7_	30.3

^a^ Means of the first ten 500 bp bins relative to the SNP – SNP ${\mathrm{r}}^{2}$ [%] ± standard deviations [%]

^b^ Means of the first ten 500 bp bins relative to the SNP – SNP ${\mathrm{r}}_{\mathrm{S}}^{2}$ [%] ± standard deviations [%]

^c^ Difference between relative ${\mathrm{r}}^{2}$ and relative${\mathrm{r}}_{\mathrm{S}}^{2}$

### Effect of allele frequency

Figure 2 revealed differences in the MAF spectra of the variant types. We therefore further evaluated local MAF differences (ΔMAF) within-population by comparing ΔMAF for the SNP – SNP and SV – SNP pairs within 5 kb distance. This revealed elevated ΔMAF for DUP – SNP, INV – SNP, and BND – SNP pairs compared to SNP – SNP and DEL – SNP pairs in BL and WL (Figs. S7, S8), but not in BR (Fig. S6). As the upper bound of ${\mathrm{r}}^{2}$ directly depends on ΔMAF [20], we investigated which part of the observed differences in the LD decay curves is due to the observed allele frequency differences. For this, we used the standardized squared correlation coefficient (${\mathrm{r}}_{\mathrm{S}}^{2}$), which expresses ${\mathrm{r}}^{2}$ as the proportion of the maximum possible ${\mathrm{r}}^{2}$ given ΔMAF of the two variants [20] and thereby excludes effects of different allele frequencies on ${\mathrm{r}}^{2}$. Mean ${\mathrm{r}}_{\mathrm{S}}^{2}$ values (Fig. S1) were generally higher than mean ${\mathrm{r}}^{2}$ values (Fig. 3) due to the removal of the allele-frequency-dependent component. While the ${\mathrm{r}}_{\mathrm{S}}^{2}$ values of DEL – SNP relative to the SNP – SNP values (Table 1) were on a comparable level of > 94% as the relative ${\mathrm{r}}^{2}$ values (-3.8% to + 0.7%), the relative ${\mathrm{r}}_{\mathrm{S}}^{2}$ values of DUPs, INVs and BNDs were between 14 and 33% higher than the according relative ${\mathrm{r}}^{2}$ values. The relative ${\mathrm{r}}_{\mathrm{S}}^{2}$ values for the complete range of 100 kb are shown in Fig. S9.

### Absence of homozygous SV genotypes

**Fig. 4:**
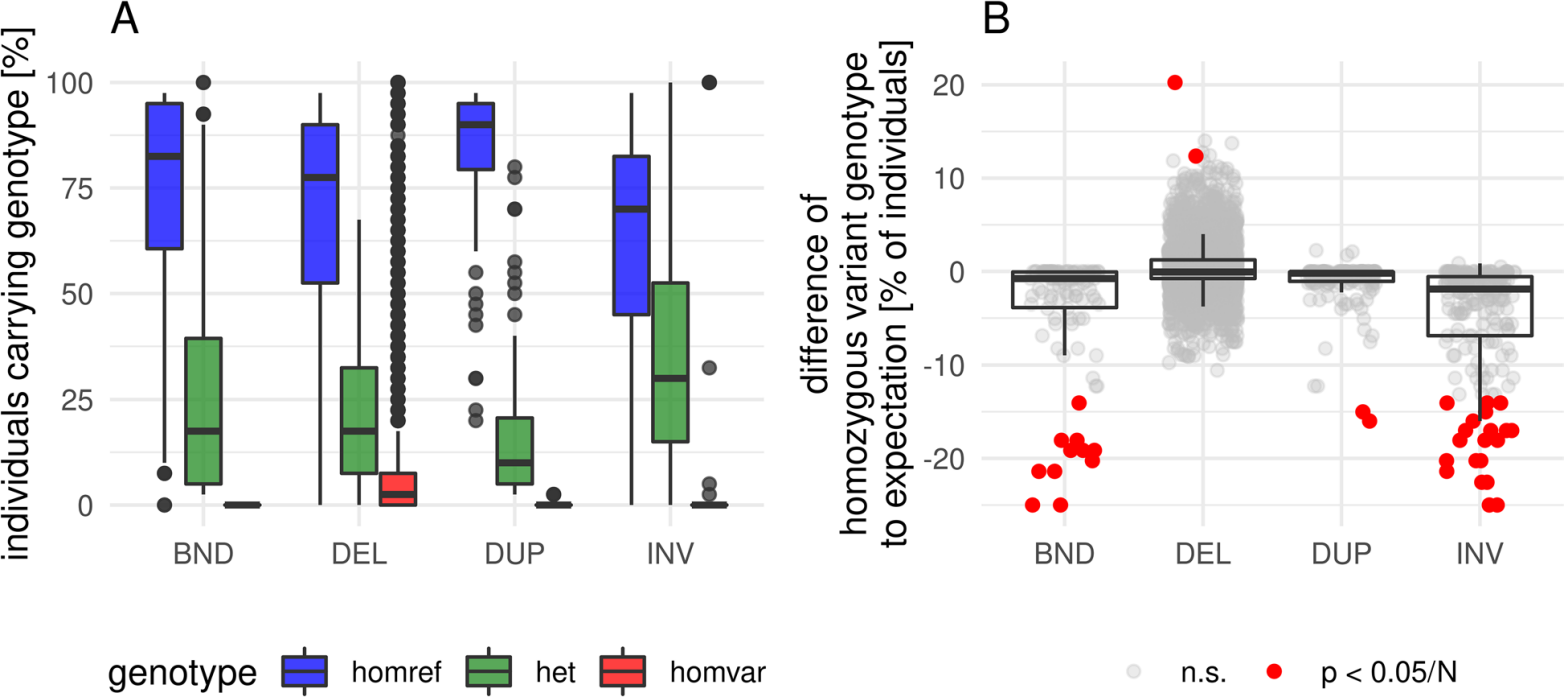
Percentage of individuals carrying SV genotype (**A**) and deviations of homozygous variant genotypes from the Hardy–Weinberg-Expectation (**B**) in the broiler population for each called SV. Deviations from HWE were tested by a Haldane Exact test under usage of the R package HardyWeinberg 1.7.2 [56]. Bonferroni correction of the p values was applied within SV class. Homref – homozygous for the reference allele; het – heterozygous; homvar – homozygous for the variant allele; n.s. – not significant. Comparable figures for WL and BL can be found in Figs. S11 and S12

During the investigation of the reasons for the lower level of LD between non-DEL SVs and SNPs, we realized a strong absence of homozygous calls for DUPs, INVs, and BNDs, but not for DEL (exemplarily demonstrated for BR in Fig. 4A). To check whether this deviation is due to small variant allele frequencies, we calculated the deviation to Hardy–Weinberg-Equilibrium (HWE) and tested those for significance, using a Haldane Exact test under usage of the R package HardyWeinberg 1.7.2 [56] (exemplarily shown for BR in Fig. 4B). Homozygous DEL calls deviated into positive as well as into negative direction from the HWE. Homozygous calls for the other SV classes instead nearly exclusively deviated into a negative direction for all populations and only negative deviations were significant.

We tried to tackle the effect of this problem by correlating the 0/1/2 coded SNP genotypes with a coverage-dependent measure of copy number for DELs and DUPs, the Duphold Flanking Fold Change (DHFFC) [57]. However, as the DHFFC was also used for filtering, the results of this are potentially confounded and are only part of the supplementary material (Supplementary File 1).

### Taggability

**Fig. 5:**
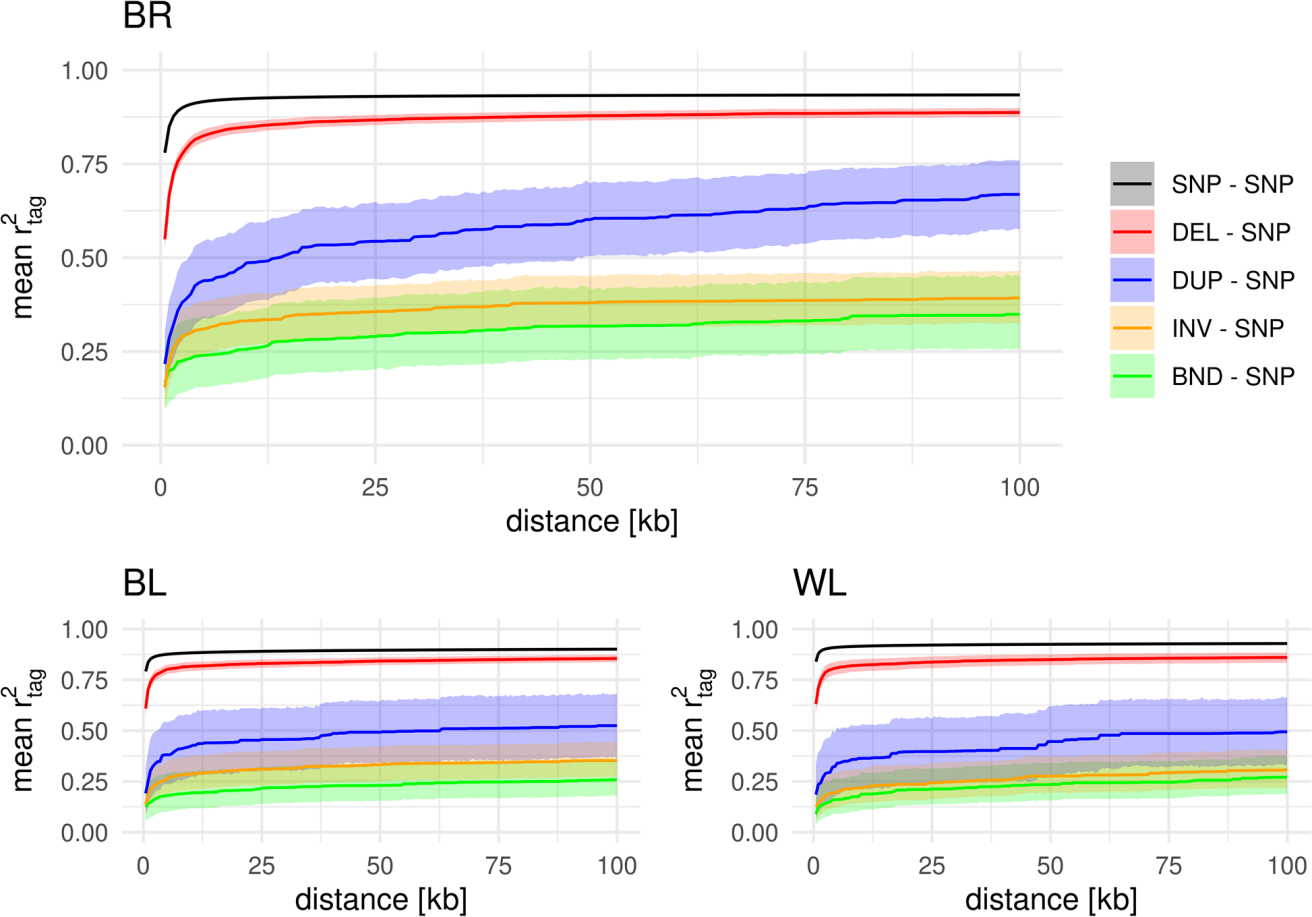
Mean taggability for broiler (BR), brown layer (BL), and white layer (WL) chickens. Taggability (${r}_{tag}^{2}$) was calculated as the maximum ${r}^{2}$ value up to a certain distance from the variant of interest. Means across variants are presented as lines while the shaded area represents the Bonferroni-corrected 95% bootstrap confidence intervals

Theoretically, one SNP in strong LD to the variant of interest would be enough to serve as a marker that (partly) captures the effect of the variant for, e.g., GWAS or genomic selection as tag SNP. We, therefore, investigated the presence of potential tag SNPs close to the variants of interest. The used measure was the maximum observed ${\mathrm{r}}^{2}$ between a variant of interest and a pool of potential tag SNPs within a certain distance (${\mathrm{r}}_{\mathrm{tag }}^{2}$). Nearly all variants in all variant classes came with at least one variable SNP within proximity of 10 kb (Fig. S14). Mean ${\mathrm{r}}_{\mathrm{tag }}^{2}$ for all variants and populations showed an asymptotic trend with identifying the best tag SNP within 10 kb for most of the variants in all three populations (Fig. 5). Only mean ${\mathrm{r}}_{\mathrm{tag }}^{2}$ of DUPs in BR was continuously growing until 100 kb distance (Fig. 5). Mean ${\mathrm{r}}_{\mathrm{tag }}^{2}$ for SNPs only reached ~ 0.9 within 100 kb in all three populations, meaning that some SNPs were not in full phase to any other SNP. Mean ${\mathrm{r}}_{\mathrm{tag }}^{2}$ was slightly reduced for DELs and strongly for DUPs, INVs and BNDs compared to SNPs (Fig. 5).

We additionally defined a variant as tagged if ${\mathrm{r}}_{\mathrm{tag }}^{2}$> 0.75 and evaluated shares of accordingly tagged variants. While more than 85% of the SNPs were tagged in BR within 10 kb, this number was slightly smaller for DELs (> 75%). More than 25% of the DUPs were tagged within 10 kb distance and 50% within 100 kb, while less than 15% of INVs and BNDs were tagged. The tendency is the same in the two layer populations, but the absolute numbers slightly deviate. As a maximum value of a sample is not independent of the number of sampled values, we also checked the number of present potential tag SNPs within 5 kb distance to the variant of interest. Interestingly, SNPs were surrounded by significantly more close variable SNPs on average than SVs in all three populations (Table 2). This difference was still present when regarding only tag SNPs (${\mathrm{r}}_{ }^{2}>$ 0.75).

**Table 2:** Median number of variable SNPs within 5 kb distance to variants of interest

Variant	BR		BL		WL	
	All	${r}^{2}\ge 0.75$	all	${r}^{2}\ge 0.75$	all	${r}^{2}\ge 0.75$
**SNP**	140 ^a^	7 ^a^	85 ^a^	5 ^a^	73 ^a^	9 ^a^
**DEL**	70 ^d^	5 ^b^	41 ^c^	4 ^b^	38 ^c^	6 ^b^
**DUP**	78 cd	4 ^b^	48 ^bc^	3 ^ab^	31 ^c^	4 ^ab^
**INV**	90 ^c^	3 ^b^	49 ^bc^	5 ^ab^	42 ^bc^	11 ^ab^
**BND**	119 ^b^	6 ^ab^	59 ^b^	1 ^ab^	61 ^b^	1 ^ab^

*Different lowercase letters within columns account for significantly different medians at the significance level of 0.05 (Bonferroni-corrected pairwise Wilcoxon rank-sum test)*

In practice, the interest of researchers and breeding companies may not be the taggability of SVs by WGS SNPs, but by array SNPs. Those come with a different allele frequency spectrum and lower resolution than WGS SNPs, which influences the LD patterns [19]. However, they are often available for a huge number of phenotyped individuals due to their use in routine breeding programs. We, therefore, evaluated the potential performance of four publically available chicken genotyping arrays with resolutions of 600 k [58], 60 k [59], 55 k [60], and 10 k [61].

The availability of variable SNPs close to the variants of interest was strongly dependent on the resolution of the arrays. While the 600 k array had a variable array SNP within 15 kb for more than 90% of the variants in all three populations, the 60 k and the 55 k array came with a slight shift of this dependency of having a variable array SNP for > 80% of the variants at 50 kb and > 90% at 100 kb (Fig. S16). The 10 k array, however, contained no variable array SNP for 50% of the variants within 100 kb. A non-random difference in SNP density by variant type is not present for any array. The reduced density compared to WGS also reduced the taggability. Mean ${\mathrm{r}}_{\mathrm{tag }}^{2}$ values for SNPs and DELs reached between 0.06 for BR and the 10 k array and 0.65 for WL and the 600 k array within 100 kb distance (Fig. S15). Interestingly, DELs seem to be slightly stronger tagged than SNPs in BL and WL (Fig. S15), while the other variant types were tagged by maximally 50% of the level which was reached in SNPs and DELs. The results are comparable when checking the proportion of variants with ${\mathrm{r}}_{\mathrm{tag }}^{2}$> 0.75 (Fig. S17). 40% of the WGS SNPs and even 45% of DELs were tagged with more than ${\mathrm{r}}_{\mathrm{tag }}^{2}$> 0.75 by a SNP of the 600 k array in WL. In contrast, less than 1% of SNPs and DELs were tagged by a SNP of the 10 k array in BR.

## Discussion

Strong LD between genomic markers and causal genomic variants is the fundamental requirement of methods like genomic prediction [62] and GWAS [63]. A stringent evaluation of LD between SNP marker panels and potentially causal SVs of different classes is therefore of strong interest for researchers and practical breeders, especially as the strength of this LD is discussed differently in literature (e.g. [6, 21–28, 32]). We here present the first study that performed this evaluation in chickens.

### Implications from the SV calling pipeline

The median sequencing coverage of the samples (5 – 17 X) was comparably low for SV discovery. Despite the fact that the sequencing depth differed between layers and broilers, results were similar for all three populations. An effect of the sequencing depth on the results is therefore unlikely, as the results could be repeated across sequencing depths.

The SV calling approach was intended to return highly accurate variant calls, therefore prioritizing precision over sensitivity. This especially required the exclusion of regions with unusually high coverage, as they may be artefacts of inaccurate read mapping in regions of low sequence complexity [64]. As those regions are known to be hot spots for SV formation by non-allelic homologous recombination (NAHR) [28–30, 65], we expect to have missed a significant proportion of SVs, especially multi-copy DUP. Further, there was a missing overlap between DELLY and MANTA at INS calling, resulting in no INS calls. A generally weak power in INS calling from short reads is expected, though [14]. Those two problems highlight the need for long-read sequencing data for future studies, which should allow for improved resolution of complex regions and comes with improved abilities for INS calling [1, 15]. The limitations of the calling approach and the resulting characteristics of the callset need to be considered when comparing our results to SV callsets that were derived by different approaches and therefore probably capturing SVs with different properties.

We further identified a lack of homozygous calls of DUPs, INVs, and BNDs with regard to HWE (Figs. 4, S11, S12). One possible reason may be a deleterious load and therefore purifying selection on those variants. While literature highlights the deleterious potential of DELs, INVs, and BNDs [2, 66], DUPs are rather considered positive by increasing gene expression [2, 3]. In our case, DELs rather show a slight excess of homozygotes than an expected lack under purifying selection (Figs. 4, S11, S12). The lack of homozygous calls was instead present for DUPs, INVs, and BNDs. Additionally, VEP impact predictions classified 99.6% of the DEL impacts as MODIFIER and only 0.4% as HIGH, while DUP impacts were classified as HIGH in 10% of the cases. The discrepancy with literature for DELs may partly be due to past inbreeding in the populations [52, 67], which resulted in small effective population sizes [68] and therefore may have purged strongly deleterious DELs [69, 70]. Purging of deleterious DELs may, together with limitations of the used SV callers, also be a reason for the relatively short sizes of the called DELs. Nevertheless, as none of the INVs and BNDs had predicted impacts besides MODIFIER, a second reason seems to be more likely: There may be deficits of the genotypers in accurately distinguishing between heterozygous and homozygous calls of DUPs, INVs, and BNDs.

### LD decay results

The overall levels of SNP – SNP LD within the populations reflect the knowledge from the literature [19, 68] and the different levels of variability (BR > BL > WL) [52, 71]. This resulted in WL having the strongest overall level of LD and BR the weakest. Besides that and if not especially indicated differently, results were the same for all three populations throughout the following sections.

The DEL – SNP LD, all in all, was on the same level as SNP – SNP LD. This implies good predictability of DEL effects by SNP call sets and is in accordance with the majority of the existing studies [6, 21–24]. Studies that found DEL – SNP LD to be on a reduced level compared to SNP – SNP LD mostly performed the DEL calling from SNP arrays, which implies low breakpoint resolution [32]. It is also common to merge CNV to copy number variable regions (CNVR) in SNP array or read-depth-based studies [32]. Therefore, a CNVR can reflect multiple mutation events and not only a single variant, resulting in reduced LD to bivariate SNPs, an effect we do not expect to be present in our data due to the more precise variant definition.

The level of DUP – SNP LD was strongly reduced compared to SNP – SNP LD and DEL – SNP LD, which is in accordance with the existing studies [22, 26, 28, 32]. However, levels of ~ 40% of the SNP – SNP LD (Table 1) were higher than what was found e.g. by Lee et al*.* [32], who found DUP – SNP LD to be ~ 20% of SNP – SNP LD in two dairy cattle populations. A main factor of DUP – SNP LD being reduced compared to SNP – SNP LD may be due to the lower allele frequencies of DUP in our callset (Fig. 2) and therefore increased local ΔMAF (Figs. S7, S8) in BL and WL. Removing the ΔMAF dependent part of LD by expressing LD as ${\mathrm{r}}_{\mathrm{S}}^{2}$ increased the relative ${\mathrm{r}}^{2}$ of 30% to a relative ${\mathrm{r}}_{\mathrm{S}}^{2}$ of 68% of the SNP – SNP ${\mathrm{r}}_{\mathrm{S}}^{2}$ (+ 28%, Table 1). This means that local differences in the allele frequency spectra between SNPs and DUP account for ~ 50% of the difference between SNP – SNP LD and DUP – SNP LD.

A second cause for reduced DUP – SNP LD could be a higher rate of genotyping errors in DUP. In fact, we identified a significant reduction of homozygous DUP calls compared to HWE (Figs. 4, S11, S12) as already discussed above. The potential genotyping inaccuracy may additionally be supported by the, admittedly subjective, observation of the two assessors during the visual filtering step that DUP came with less clear support than DEL. This, however, resulted only in a moderately reduced inter-observer reliability of 94% in DUP compared to 97% in DEL (Supplementary file 3).

A further possibility of reduced DUP – SNP LD may be the occurrence of multi-copy CNVs (mCNVs) [28, 30] in our callset. DUP in the callset may partly represent CNVs that occur with different copy numbers and are therefore multi- instead of bivariate variants. This reduces the linkage to bivariate SNPs. We saw slight support for the occurrence of some mCNV in the callset e.g. by some high DHFFC values. However, mCNVs are known to cluster in special regions of the genome [28] due to non-allelic homologous recombination (NAHR) as a formation mechanism [29, 72]. Note that NAHR can also occur recurrently [29], resulting in variants that are called bivariate but stem from multiple mutation events. As those clusters should result in high-coverage regions, which we removed in the filtering step, we do not expect a higher number of mCNV and recurrent mutations in our callset.

We also evaluated the linkage between SNPs and INV/ BND and found low levels of LD (26.8% and 18.5% of SNP – SNP LD). The reduced LD in our study is again partly due to local allele frequency differences (Figs. S6— S8) as for DUP. Relative ${\mathrm{r}}_{\mathrm{s}}^{2}$ values were therefore 14% to 30% higher than relative ${\mathrm{r}}^{2}$ values (Table 1). However, ${\mathrm{r}}_{\mathrm{s}}^{2}$ values for INV – SNP and BND – SNP were still only ~ 50% of SNP – SNP ${\mathrm{r}}_{\mathrm{s}}^{2}$. The remaining gap may partly be due to genotyping problems. We identified the lack of homozygous calls for INVs and BNDs (Figs. 4, S11, S12) as for DUPs. In combination with the missing ability to use coverage information for filtering, we would trust the INV and BND genotypes least in our callset. In contrast to our results, Sudmant et al*.* [28] found INV to be in good LD to SNPs in a very accurate callset from 2,504 human genomes, which further supports that the accuracy of INV calls was low in our study.

### Taggability

The analysis of taggability revealed comparable patterns as the LD decay. A high fraction of SNPs and DEL was tagged by close-by WGS SNPs in all three populations (Figs. 5; S14), while only a small fraction of DUPs, INVs, and BNDs was tagged. However, in contrast to the decay patterns, SNPs on average were tagged slightly stronger than DEL, and between 5 and 10% more SNPs were tagged with ${\mathrm{r}}_{\mathrm{tag}}^{2}$ > 0.75 than DEL. A reason for the higher taggability of SNPs compared to DEL, while the LD decay does not differ, may be the reduced SNP density around DELs (Table 2), as the chance for higher maximum values increases with the number of SNPs in the region of interest. In contrast, DELs were tagged slightly better by array SNPs than WGS SNPs by array SNPs. In the case of array SNPs, no locally increased density was present, as array design aims at an equidistant spacing of markers across the genome [58]. This resulted in no difference between the taggability of SNPs and DELs by array SNPs. Potential issues of excluding SNPs in complex regions during array design as suggested by Lee et al*.* [32] as a reason for reduced CNV – SNP LD, were not observed in this study, as we excluded SVs in those regions due to a minor calling accuracy. Using array SNPs to tag the WGS variants further revealed a strong need for dense marker maps to provide good tag SNPs, as only the 600 k array could provide tag SNPs with ${\mathrm{r}}_{\mathrm{tag}}^{2}$ > 0.75 for more than 25% of SNPs and DEL. This may largely explain why e.g. Xu et al*.* [33] found a quarter of CNVs that were significantly associated with milk traits in Holstein cattle to be not tagged by SNPs of a 50 k array. It suggests that this is not solely due to the nature of CNV but that they also missed a comparable fraction of effects, which are caused by SNPs.

The concept of taggability is especially relevant for GWAS, where phenotype-marker associations are tested for each marker separately. The strength of the LD between marker and causal variant then directly influences the power of the GWAS. However, the absence of single tag SNPs does not imply that the effect of an SV cannot be captured by a longer haplotype. Methods that utilize effects of multiple SNP at once (e.g. ridge regression best linear unbiased prediction [62]), of which each can explain a slightly different fraction of the variance of the causal variant, may be more robust in this sense. Additionally, imputation of known SVs would probably be a way to overcome the issue of low taggability and needs further investigation.

## Conclusions

We evaluated LD patterns between a comprehensive SV callset and surrounding SNPs in three commercial chicken populations. We found DEL – SNP LD to be on the same level as SNP – SNP LD, while DUP – SNP, INV – SNP, and BND – SNP LD were strongly reduced. This was in accordance with the availability of tag SNPs for a high share of SNPs and DELs, while tag SNPs for DUPs were rare and mostly missing for INVs and BNDs. Different arrays came with a density-dependent ability to tag WGS SNPs and SVs but did not show strong systematic differences compared with taggability by WGS SNPs. The main reason for existing differences in SNP – SNP and DUP/INV/BND – SNP LD in our study was due to local MAF differences. Those accounted for ~ 50% of this difference in the strength of LD. This implies that genomic variance due to DELs in the chicken populations studied can be captured by different SNP marker sets as good as variance from WGS SNPs, whereas separate SV calling might be advisable for DUP, INV, and BND effects.

## Material and methods

### Data

The study used WGS data of 25 white layers, 25 brown layers, and 40 broiler chickens. The raw data was first published by Qanbari et al*.* [52], which contains more information about the samples. Chickens were paired-end sequenced with a median coverage between 5 and 17 X, read length of 100 bp (WL + BL) or 126 bp (BR), and insert sizes of ~ 400 bp. Basic quality statistics can be found in Supplementary file 2 as MultiQC report [73].

Population integrity was controlled using principal component analysis in plink 1.9 [74]. The SNPs were first LD pruned by setting the –indep-pairwise flag to sliding windows of 50 kb, a stepsize of five SNPs and an ${\mathrm{r}}^{2}$ of 0.5. Based on the pruned SNPs, plink extracted then 90 prime components. Results for the first four prime components and the variance explained can be found in Fig. S18. The first two prime components, which in total accounted for 33.2% of the total variance, clearly separated broilers, white- and brown layers. The two broiler subpopulations were only slightly separated by the second prime component and clearly by the third, which accounted for 4.5% of the total variance. The fourth component started splitting one of the broiler populations. We assumed this to be sufficiently closely related to consider the two broiler subpopulations as a combined population for further analyses.

### Variant calling pipeline

Alignment on the reference genome galGal6/ GRGC6a and SNP calling were conducted in a previous study [53] following GATK best practices pipeline [75]. The SNPs needed for this study were then extracted from the old callset using bcftools [76] and the duplicate-marked and base quality score recalibrated BAM files were used as starting point for the SV calling process.

SV calling was conducted following a consensus calling approach. SVs were first separately called per individual and then genotyped on population-level by running Delly 0.8.5 [10], Manta 1.6.0 [12], and a combination of Lumpy 0.2.13 [11] and Svtyper 0.7.0 [77] in parallel on the complete set. The genotyping results of the three calling pipelines were then merged using SURVIVOR 1.0.7 [78] and allowing for breakpoint differences of 1000 bp. This resulted in 95,478 raw SV calls.

Additionally, read depth profiles for all samples in 100 bp windows were generated using Mosdepth 0.2.9 [79] and SVs were annotated with Dupholds (version 0.2.1) [57] flanking fold change (DHFFC) and the SNP genotype calls located on the SV.

The merged callset was then filtered based on the following parameters:

1. Caller overlap: At least two of the three callers needed to support the variant.
2. Genotype concordance: The genotype that was supported by two out of the three callers was considered as the consensus genotype. Genotypes without the necessary support were set to missing for later re-imputation. If more than two samples did not have the necessary genotype concordance support for an SV, the complete SV was removed from the data set.
3. Removal of high coverage regions: Local coverage was extracted by Mosdepth 0.2.9 [79] in 100 bp windows. If windows exceeded a threshold of twice the average coverage across all samples (expected value for a fixed DUP) plus two standard deviations, they were classified as unusually highly covered. Unusually highly covered regions were further merged if they were less than 1000 bp apart from each other. SVs with breakpoint confidence intervals falling in such a region were removed from the data set.
4. Difference to flanking coverage: DELs and DUPs calls were checked for non-consistent coverage changes relative to the flanking coverage by evaluating the Duphold Flanking Fold Change (DHFFC) [57]. DELs were considered as wrong genotypes when heterozygotes were not between 0.1 and 0.9 and homozygous DEL genotypes not smaller than 0.25. Heterozygous DUPs had to be > 1.1 and homozygous DUPs > 1.5. DELs/DUPs with more than one error or more than 10% wrong genotypes were filtered. Otherwise, the putatively wrong DEL/DUP genotypes were set to missing for later re-imputation.
5. Support by SNP calls on DELs: SNP calls need to be homozygous on heterozygous DELs and missing on homozygous DELs. We, therefore, calculated for each DEL genotype the relative number of wrong SNP genotypes (e.g. one error by five total SNPs on the DEL = 0.1). If the sum of those error rates across samples exceeded two or 50% of the number of samples that were at least heterozygous for the DEL, the DEL was filtered. Otherwise, the putatively wrong DEL genotypes were set to missing for later re-imputation.

This resulted in 5,600 SVs (4,831 DELs; 253 DUPs; 346 INVs; 170 BNDs; 94.1% filtered). No INS remained, as Lumpy does not call INS and there was no overlap between Delly and Manta. Samplot 1.0.19 [54] was then used to generate quality control plots for each SV that passed the previous filtering step. The quality plots were visually screened by two separate observers comparable to the workflow implemented in SV-plaudit [80], but implemented locally by using image-sorter2 (https://github.com/Nestak2/image-sorter2). The SVs needed to be scored as ‘pass’ by each of the two observers to be further used (Supplementary file 3). By this, a further 6.9% of the SVs (3.5% of DEL, 11.1% of DUP, 36.0% of INV, and 30.8% of BND) were removed. The removed SVs were mainly in regions with complex mapping patterns.

The final SV callset (4,301 DEL, 224 DUP, 218 INV, 117 BND) was then merged with the SNP callset (12,294,329 bivariate autosomal SNPs). The samples were phased and missing genotypes were imputed by beagle 5.0 [81] with default settings besides reducing ‘ne’ to 10,000 [82]. Functional consequences were annotated by ensembl-vep [55] using the release 100 GRGC6a annotation files.

### Estimation of LD

LD between two loci with a maximum distance of 100 kb was initially estimated from phased haplotypes as follows:

$${r}_{AB}^{2}=\frac{{\left({p}_{AB}-{p}_{A}{p}_{B}\right)}^{2}}{{p}_{A}{p}_{B}(1-{p}_{A})(1-{p}_{B})}$$

where ${\mathrm{p}}_{\mathrm{A}}$ and ${\mathrm{p}}_{\mathrm{B}}$ account for the alternative allele frequencies at the two loci and ${\mathrm{p}}_{\mathrm{AB}}$ for the according haplotype frequency. To control for allele frequency deviations that influence the maximum possible ${\mathrm{r}}^{2}$, we further scaled ${\mathrm{r}}^{2}$ by the maximum possible ${\mathrm{r}}^{2}$ given $\mathrm{\Delta MAF}$ (${\mathrm{r}}_{\mathrm{S}}^{2}={\mathrm{r}}^{2}/{\mathrm{r}}_{\mathrm{max}|\mathrm{\Delta MAF}}^{2}$) where ${\mathrm{r}}_{\mathrm{max}|\mathrm{\Delta MAF}}^{2}$ was derived as described by VanLiere and Rosenberg [20]. As we realized a problem with calling of homozygous DUP, we additionally estimated LD as squared Pearson Correlation between 0/1/2 coded SNP genotypes and the Duphold Flanking Fold Change (DHFFC) [57] as a measure for the relative reference genome coverage at DEL and DUP (due to possible confounding only part of Supplementary file 1). LD decay was then summarized in means of 500 bp bins between the variants.

Bonferroni corrected bootstrap confidence intervals for the LD decay were estimated by resampling the ${\mathrm{r}}^{2}$ values within each bin 100,000 times with replacement. As tests showed confidence intervals for SNP—SNP LD being < 0.001 due to the huge number of underlying values, we decided to skip estimation of confidence intervals for bins with > 1 M ${\mathrm{r}}^{2}$ values.

A tag SNP was defined as the SNP with the highest ${\mathrm{r}}^{2}$ to the variant of interest within a certain distance (${\mathrm{r}}_{\mathrm{tag}}^{2})$. The taggability of variant classes was then investigated by comparing means of ${\mathrm{r}}_{\mathrm{tag}}^{2}$ and shares of variants with ${\mathrm{r}}_{\mathrm{tag}}^{2}>0.75$. Additionally to the taggability by WGS SNPs, we compared the taggability by SNPs of four commercially available SNP arrays. The 600 k Affymetrix Axiom chicken genotyping array [58], a 60 k Illumina Bead Chip [59], a 55 k Affymetrix genotyping array [60], and the IMAGE_001 multispecies array, which contains 10 k chicken-specific SNPs on an Affymetrix genotyping array [61]. The annotation files were lifted over to the reference genome galGal6/GRGC6a by the UCSC [83] liftOver tool under usage of the according chain files and the overlaps with the variable WGS SNPs were defined as pools of potential Array tag SNPs.

### Workflow

The complete pipeline was set up in snakemake 5.3.0 [84] and the according scripts including the snakefile with all used parameters as well as the dependency analytics graph (DAG) and the rulegraph of the pipeline can be found on Zenodo (https://doi.org/10.5281/zenodo.5770348).

## Supplementary Information

**Additional file 1.** Supplementary results, tables and figures.

**Additional file 2.** MultiQC report.

**Additional file 3.** Observer concordance of the visual filtering step.

## Data Availability

The raw fastq files were already published by Qanbari et al. [52] and can be accessed via the ENA project PRJEB30270 (https://www.ebi.ac.uk/ena/browser/view/PRJEB30270). The snakemake workflow together with all scripts and supplementary files can be found on Zenodo (https://doi.org/10.5281/zenodo.5770348). Intermediate results are available from the corresponding author on reasonable request.
