# Supplementary material for: Assessment of linkage disequilibrium patterns between structural variants and single nucleotide polymorphisms in three commercial chicken populations
Source: BMC Genomics. 2022 Mar 9;23:193. doi: 10.1186/s12864-022-08418-7 (PMC8908679; doi:10.1186/s12864-022-08418-7)
Supplement: Supplementary file 1 — Additional file 1. Supplementary results, tables and figures. [file 12864_2022_8418_MOESM1_ESM.pdf]

Supplementary file 1

Supplementary results, tables and figures

Correlation between SNP genotypes and DHFFC

We checked for possible problems with the genotyping by correlating the 0/1/2 coded SNP genotypes with a coverage-dependent measure of copy number for DELs and DUPs, the Duphold Flanking Fold Change (DHFFC) [1]. The DHFFC expresses the coverage of a variant in relation to the coverage of the close surrounding genomic region (e.g. 0 = homozygous DEL, 1 = homozygous reference, 2 = homozygous DUP) and therefore allows for a genotyping-tool independent expression of the copy count of CNVs. However, as the DHFFC was already used for filtering, this analysis may be confounded. The SNP – SNP LD slightly increased for this measure (Figure S 2). While the DUP – SNP LD relative to the SNP-SNP LD (Table S 1) stayed approximately constant for BR in comparison to the haplotype-based r^2 (Table 1), it increased for BL (+7 %) and WL (+15 %). In contrast, the DEL – SNP LD in relation to SNP – SNP LD strongly decreased (-19 % – -24 %). However, the DUP – SNP LD curve did not show the expected LD decay by distance anymore but stayed constant (Figure S 2).

Supplementary tables

Table S 1: Mean squared correlation between SNP genotypes and DHFFC of DEL and DUP relative to the squared SNP – SNP correlation as the average of the first 10 500 bp distance bins

Type	All		BR		BL		WL	
	value[*]	Δ[**]	value[*]	Δ[**]	value[*]	Δ[**]	value[*]	Δ[**]
**DEL – SNP**	78.9 ± 6.9	-21.2	71.1 ± 3.0	-24.3	86.8 ± 2.1	-20.2	78.9 ± 1.6	-19.2
**DUP – SNP**	47.2 ± 8.6	7.3	39.2 ± 5.7	-0.3	48.1 ± 4.8	7.0	54.4 ± 7.1	15.3

[*]Means of first 10 500 bp bins relative to SNP – SNP r^2 [%] ± standard deviations [%]
[**]Difference to relative r^2 (Table 1)

Supplementary figures

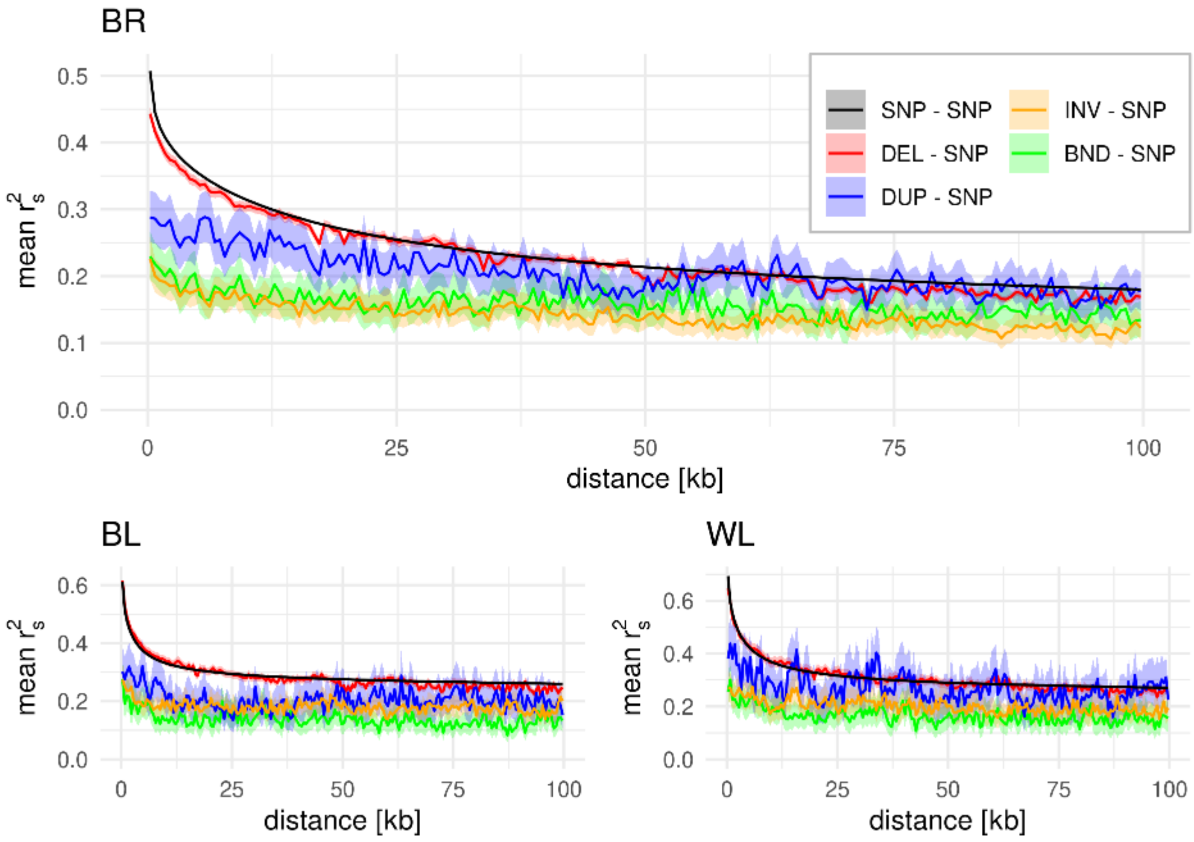

Figure S 1: Allele frequency corrected LD decay in the broiler (BR), brown layer (BL) and white layer (WL) chickens. The LD is presented as mean r_S^2 in 500 bp distance bins and the shaded areas represent Bonferroni-corrected 95 % bootstrap confidence intervals. For SNP – SNP distance bins with > 1M r_S^2 values, no confidence intervals were estimated.

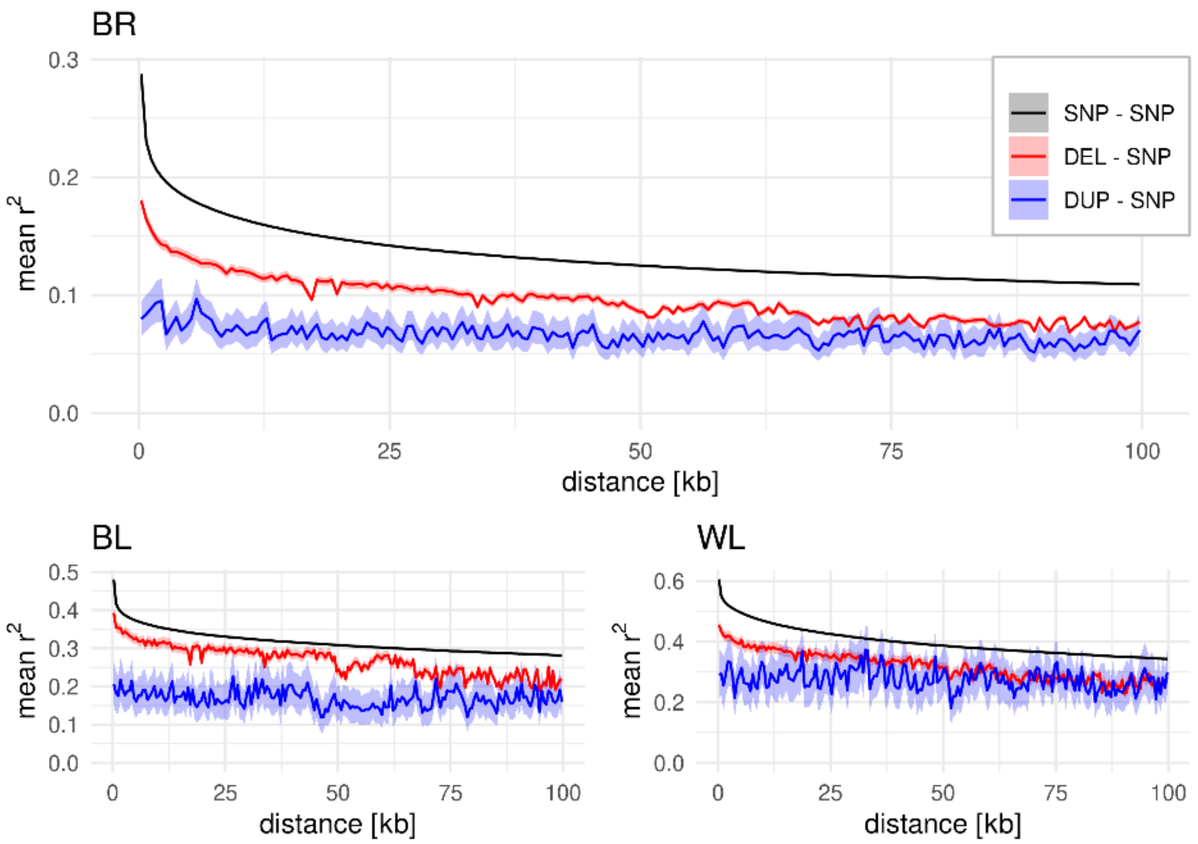

Figure S 2: LD decay for broiler (BR), brown layer (BL) and white layer (WL) chickens. LD (r^2) was calculated as the correlation between SNP genotypes and Duphold Flanking Fold Change (DHFFC) of DEL and DUP.

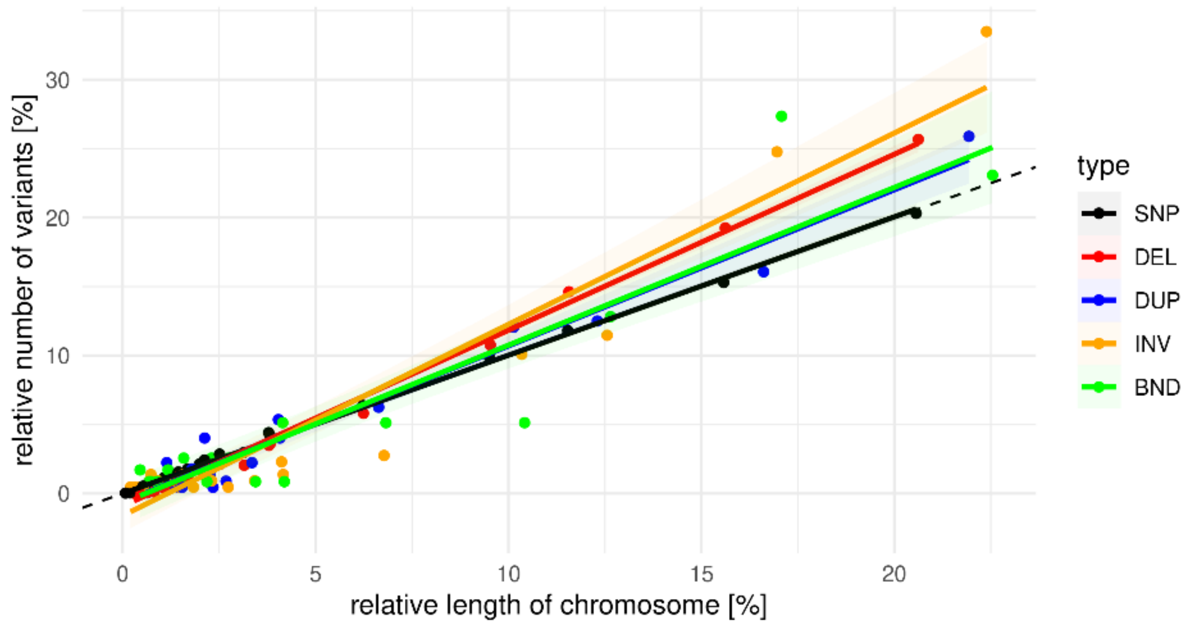

Figure S 3: Relative number of called variants by chromosome length. The dashed black line represents the line of identity, while the solid lines represent the regression of relative variant number on relative chromosome length.

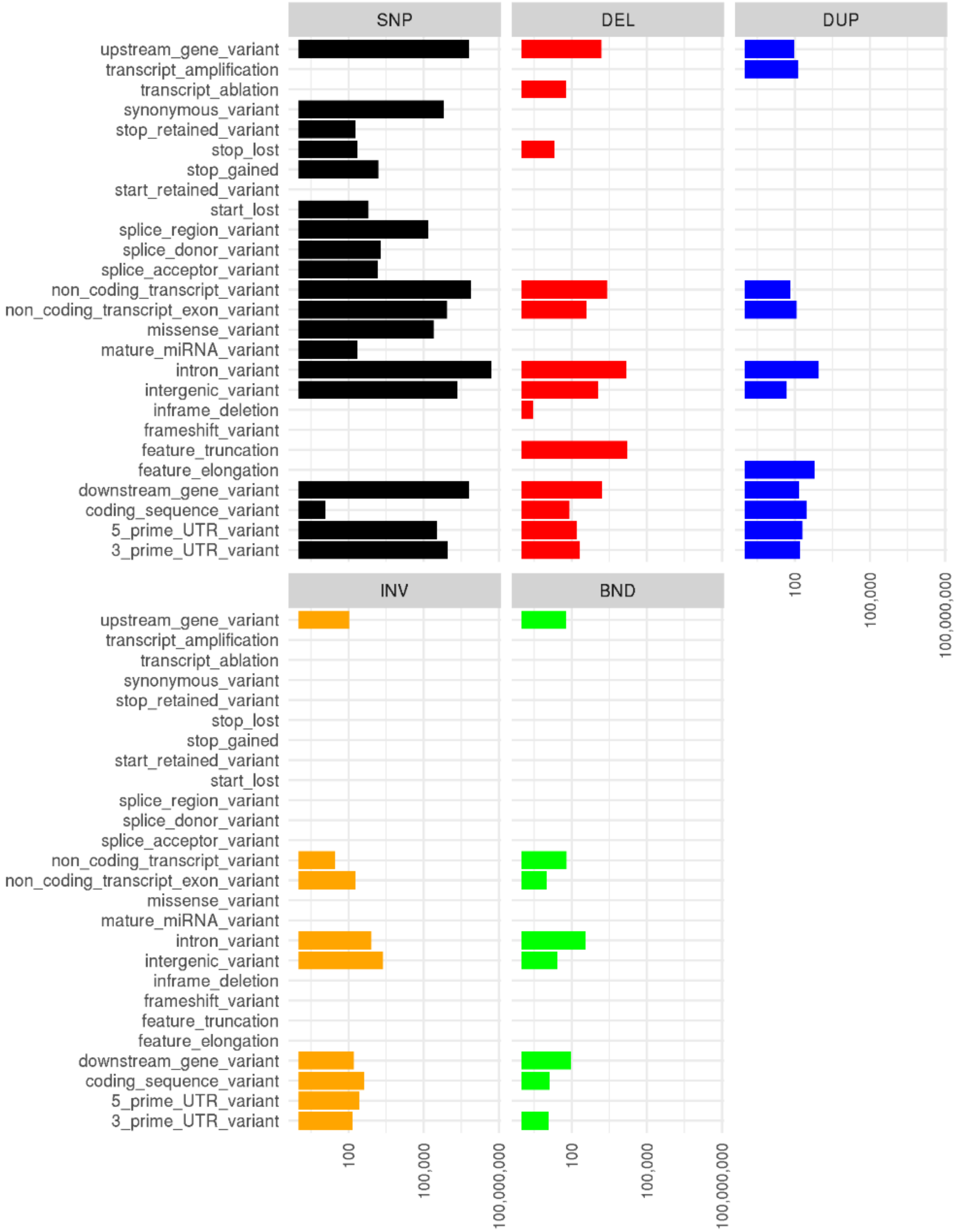

41

42 *Figure S 4: Functional consequences of the called variants. Consequences were predicted by Ensebl VEP.*
43 *Note that one variant can impact multiple genes and a gene can be impacted by multiple variants.*

44

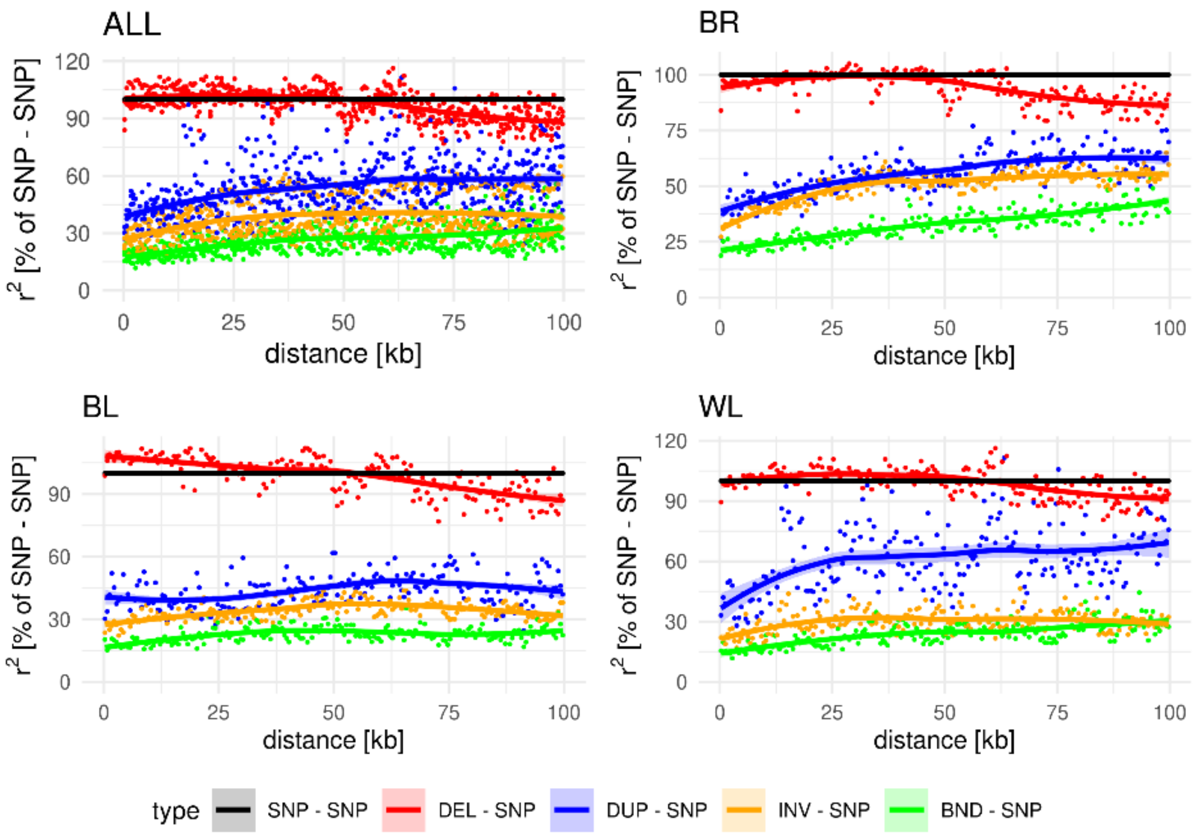

Figure S 5: Relative r^2 *values by distance, variant type and population. Values represent means of 500 bp bins. The trend is marked by smoothing lines.*

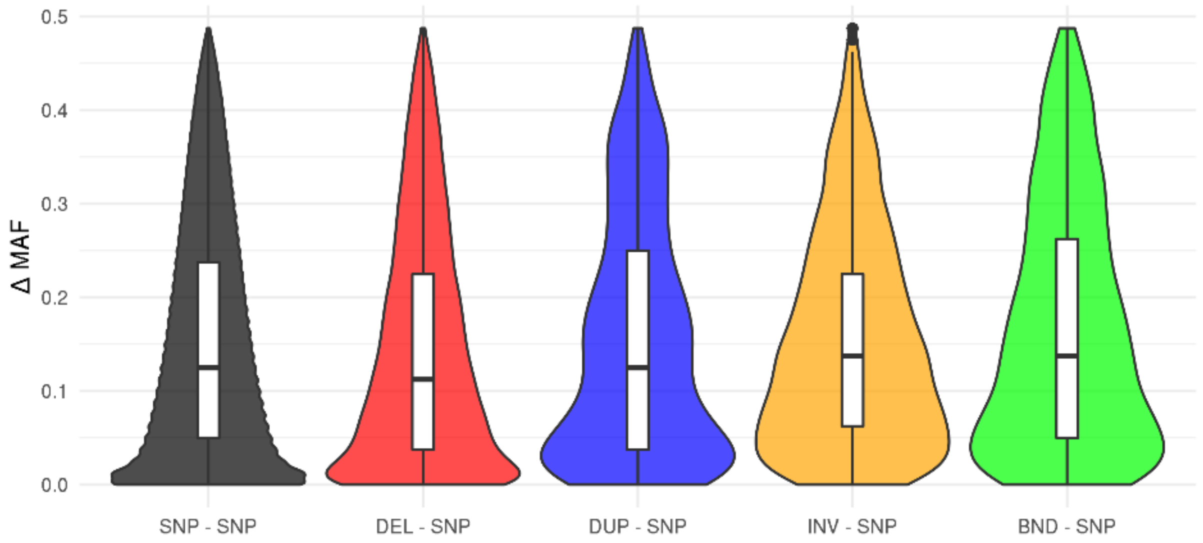

Figure S 6: Distribution of local differences in minor allele frequencies (ΔMAF) for variant pairs in broilers (BR). Pairs up to 5 kb distance were considered and SNP – SNP pairs were randomly sampled down to 1/100.

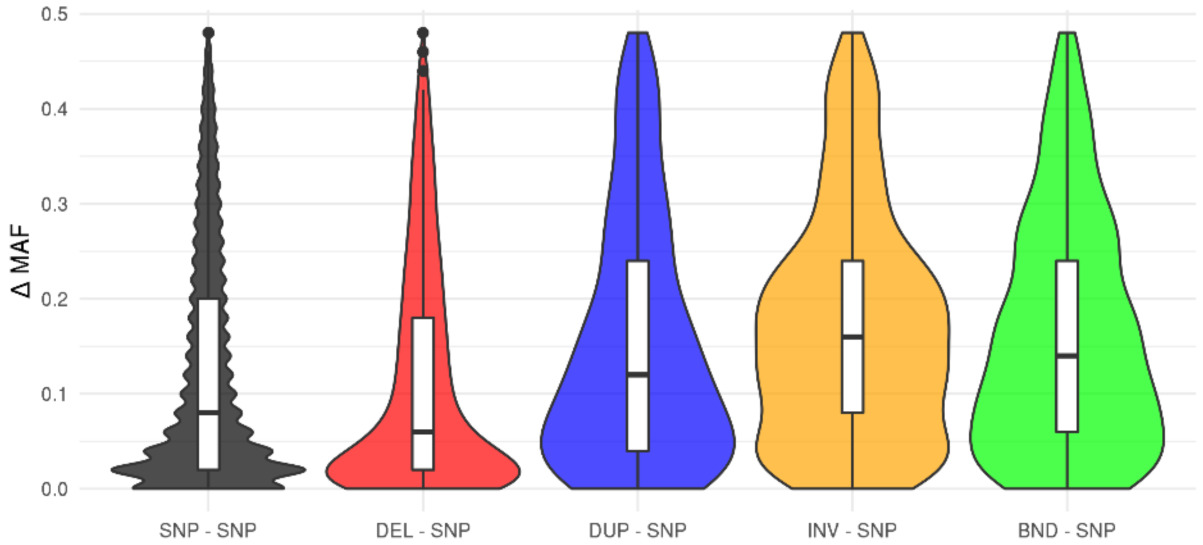

Figure S 7: Distribution of local differences in minor allele frequencies (ΔMAF) for variant pairs in brown layers (BL). Pairs up to 5 kb distance were considered and SNP – SNP pairs were randomly sampled down to 1/100.

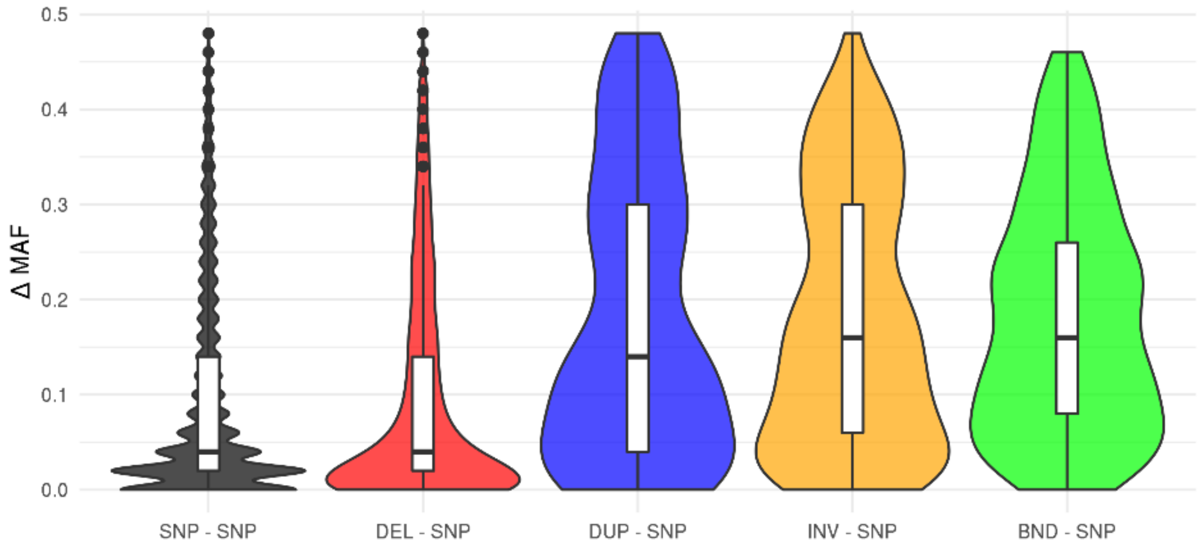

Figure S 8: Distribution of local differences in minor allele frequencies (ΔMAF) for variant pairs in white layers (WL). Pairs up to 5 kb distance were considered and SNP – SNP pairs were randomly sampled down to 1/100.

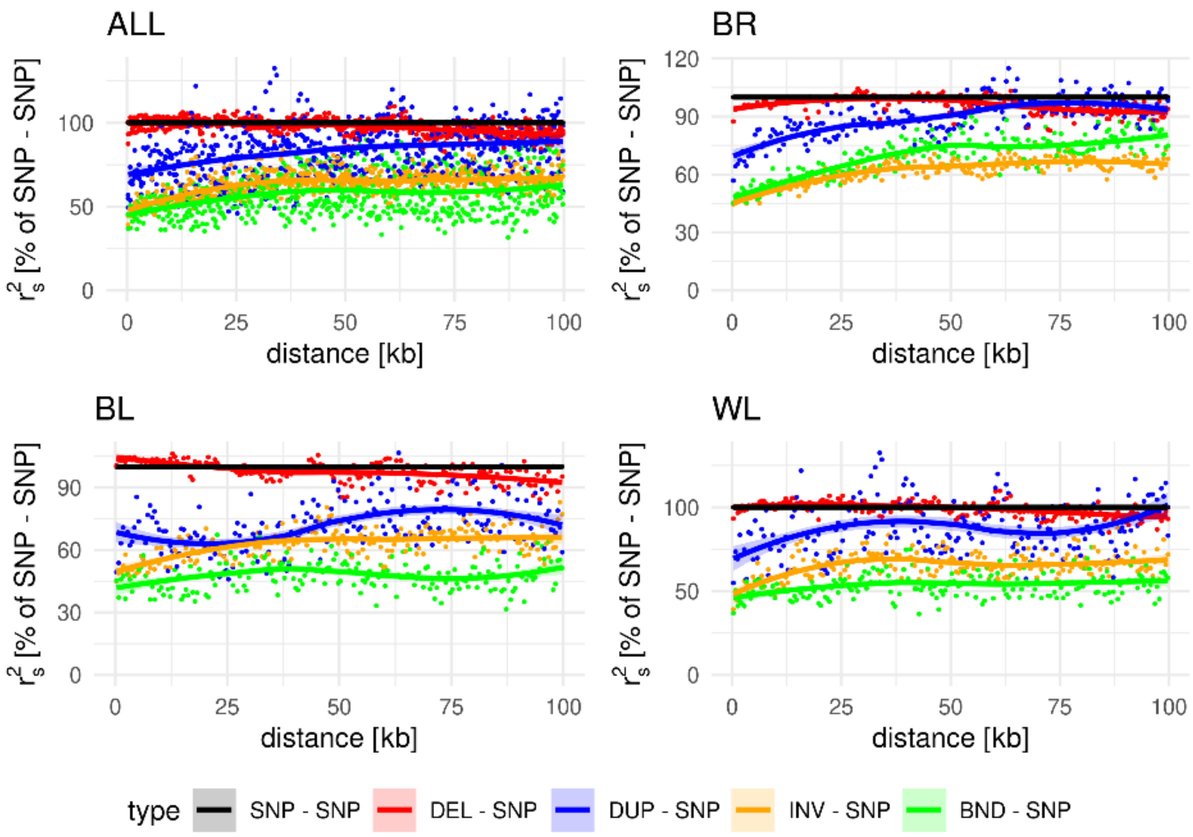

Figure S 9: Relative r_S^2 *values by distance, variant type and population. Values represent means of 500 bp bins. The trend is marked by smoothing lines.*

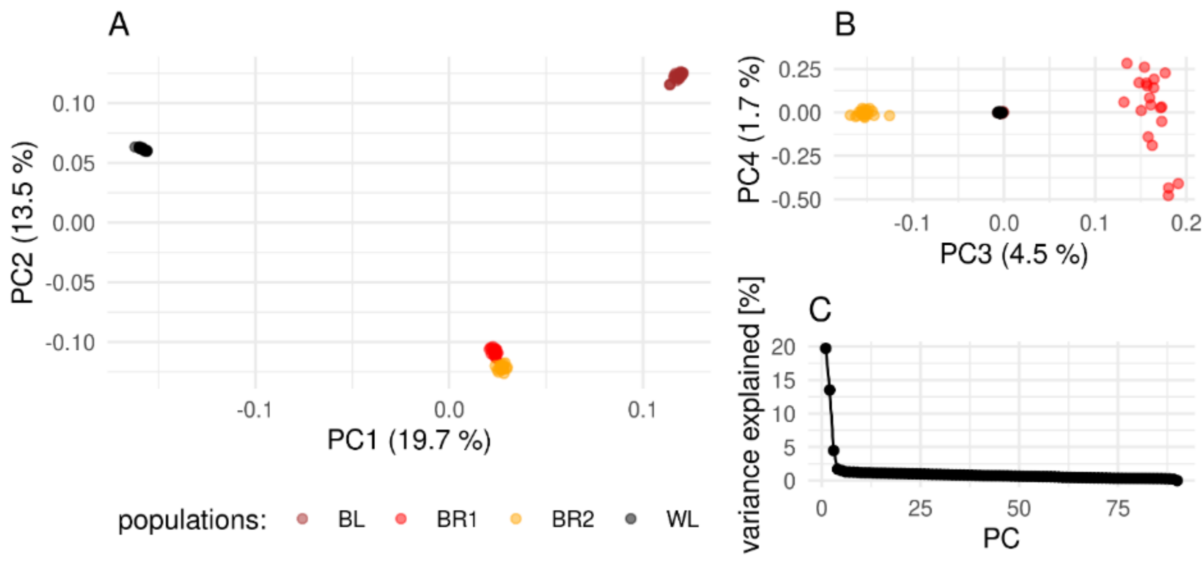

Figure S 10: Results of prime component analysis. First vs. second prime component (A), third vs. fourth prime component (B) and variance explained (C). The percentage of explained variance is denoted within brackets in the axis labels. BL – brown layer, BR1/2 – broiler, WL – white layer.

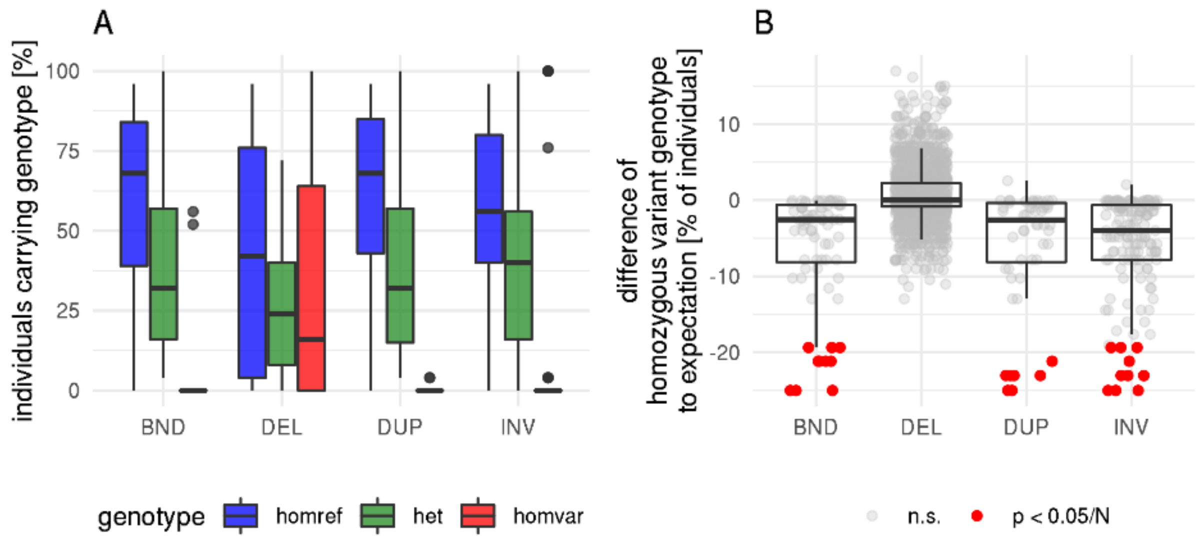

Figure S 11: Percentage of individuals carrying SV genotype (A) and deviations of homozygous variant genotypes from the Hardy-Weinberg-Expectation (B) in the brown layer population for each called SV. Deviations from HWE were tested by a Haldane Exact test under usage of the R package HardyWeinberg 1.7.2 [2]. Bonferroni correction of the p values was applied within SV class. Homref – homozygous for the reference allele; het – heterozygous; homvar – homozygous for the variant allele; n.s. – not significant.

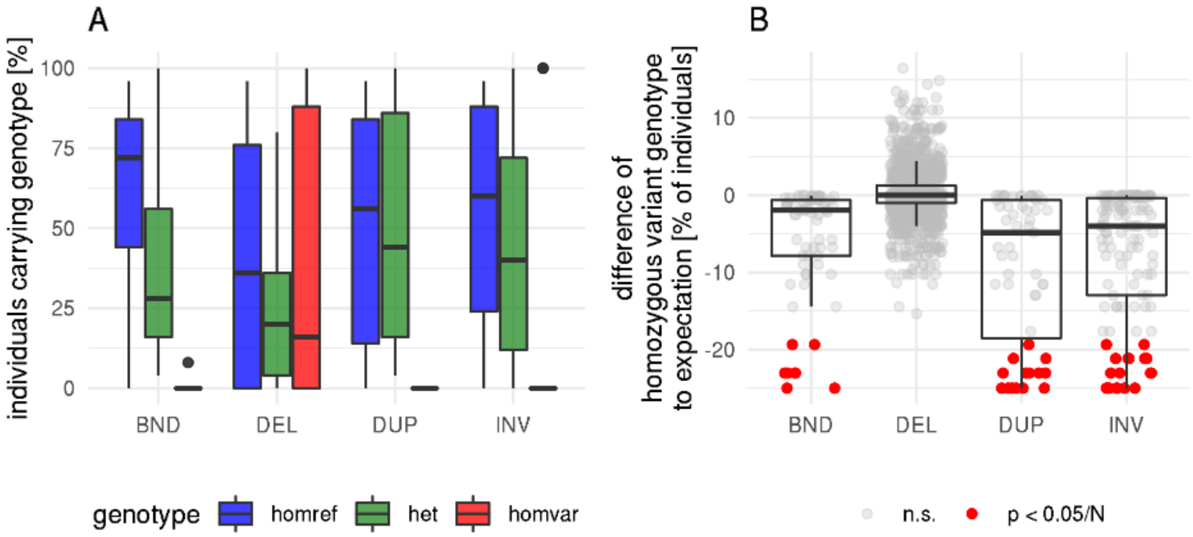

Figure S 12: Percentage of individuals carrying SV genotype (A) and deviations of homozygous variant genotypes from the Hardy-Weinberg-Expectation (B) in the white layer population for each called SV. Deviations from HWE were tested by a Haldane Exact test under usage of the R package HardyWeinberg 1.7.2 [2]. Bonferroni correction of the p values was applied within SV class. Homref – homozygous for the reference allele; het – heterozygous; homvar – homozygous for the variant allele; n.s. – not significant.

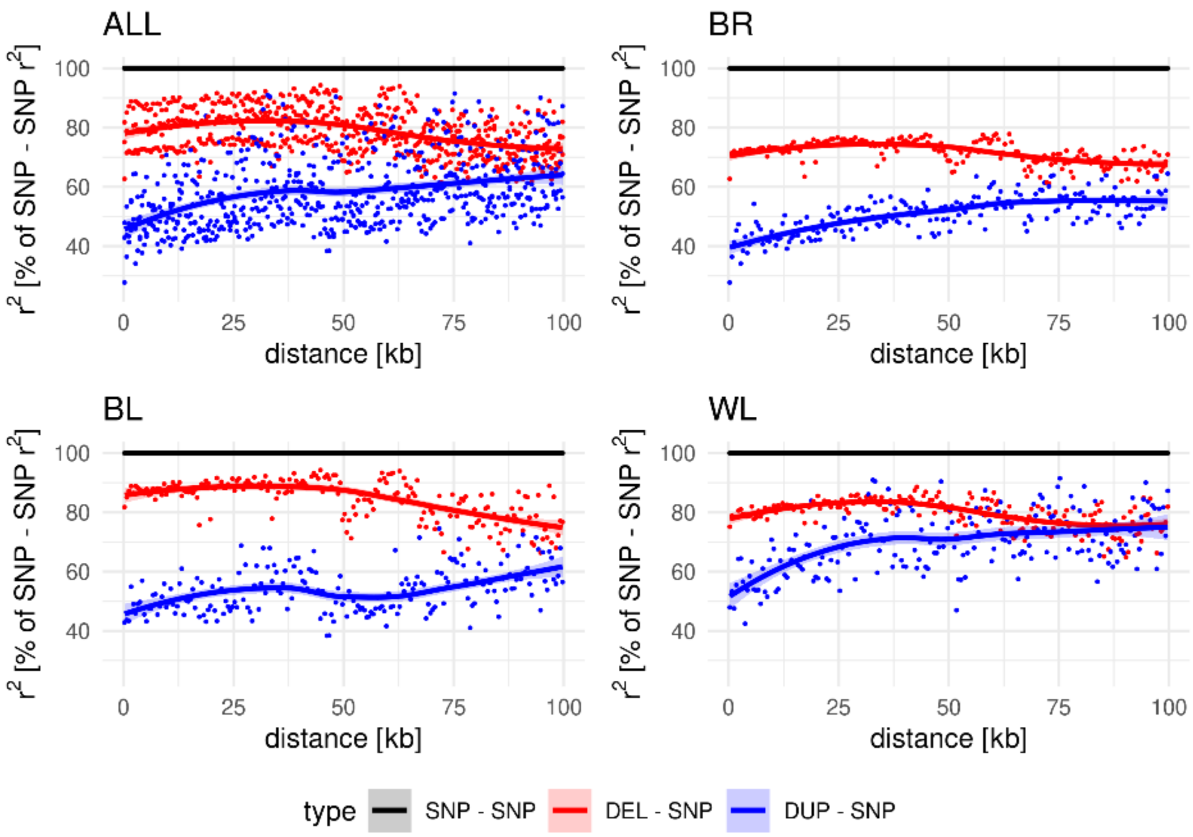

Figure S 13: Relative r^2 values by distance, variant type and population. LD (r^2) is calculated as correlation between SNP genotypes and DUPHOLD FLANKING FOLD CHANGE (DHFFC) of DEL and DUP. Values represent means of 500 bp bins. The trend is marked by smoothing lines.

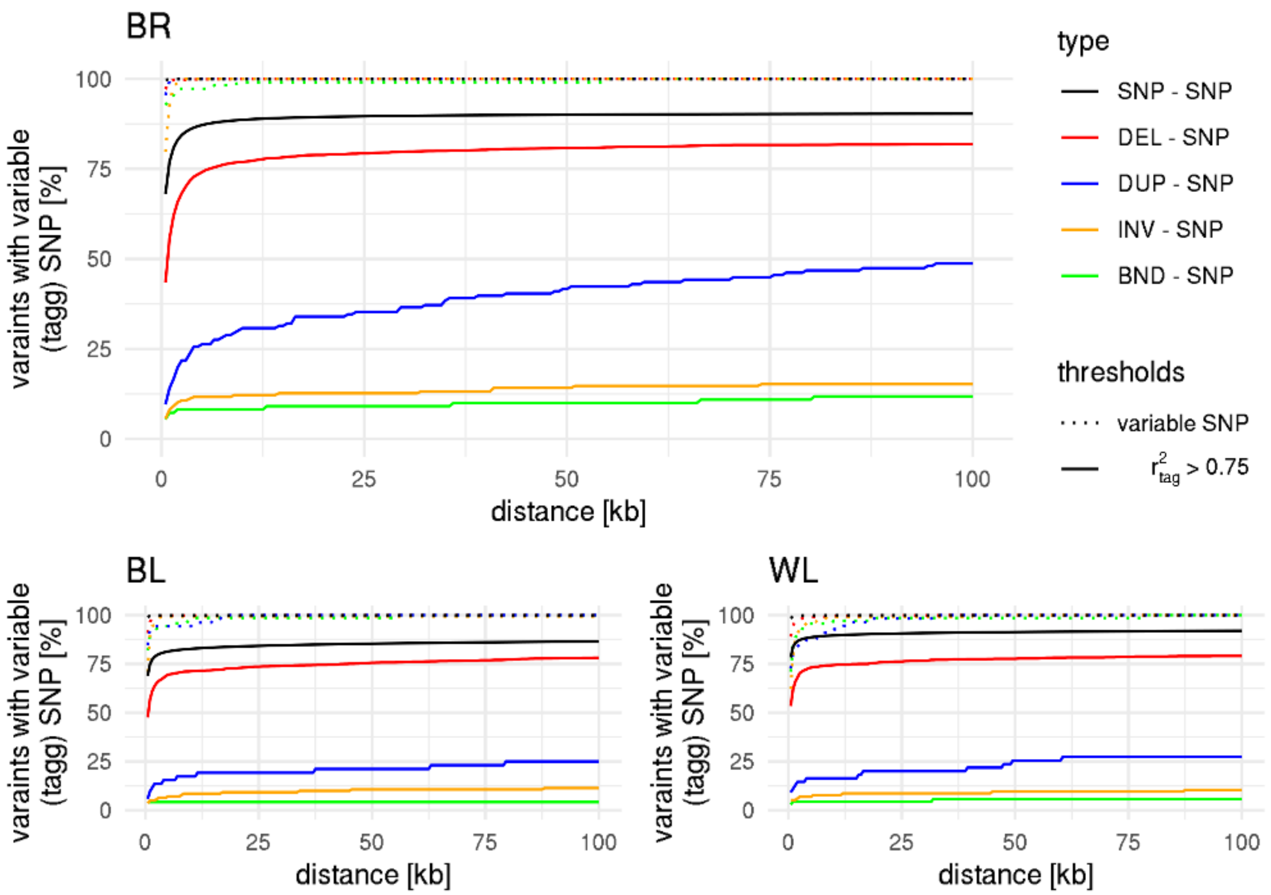

Figure S 14: Percentage of variants with variable SNP or tag SNP ($r^2_{tag} > 0.75$) for broiler (BR), brown layer (BL) and white layer (WL) chickens. Taggability was calculated as the maximum r^2 value up to a certain distance from the variant of interest.

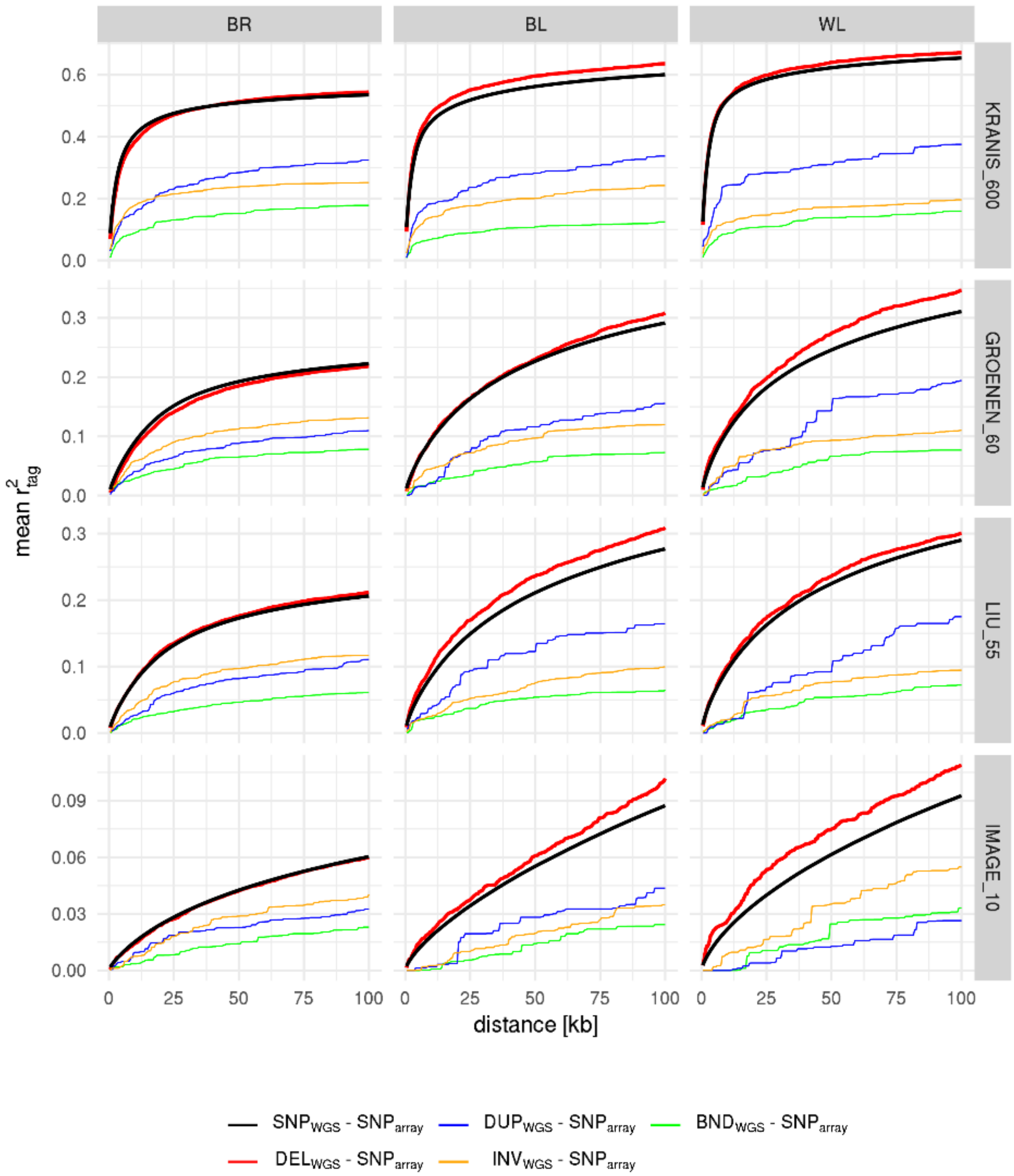

Figure S 15: Mean taggability (r^2_{tag}) between WGS variants and array SNPs by distance and variant type for broiler (BR), brown layer (BL) and white layer (WL) chickens and four different genotyping arrays. Taggability was calculated as the maximum r^2 value up to a certain distance from the variant of interest. KRANIS_600 = 600k Affymetrix array [3]; GROENEN_60 = 60k Illumina Bead Chip [4]; LIU_55 = 55k Affymetrix genotyping array [5]; IMAGE_10 = 10k Affymetrix genotyping array (IMAGE_001 multi-species array [6]). Note that the y-axis is scaled to the according array.

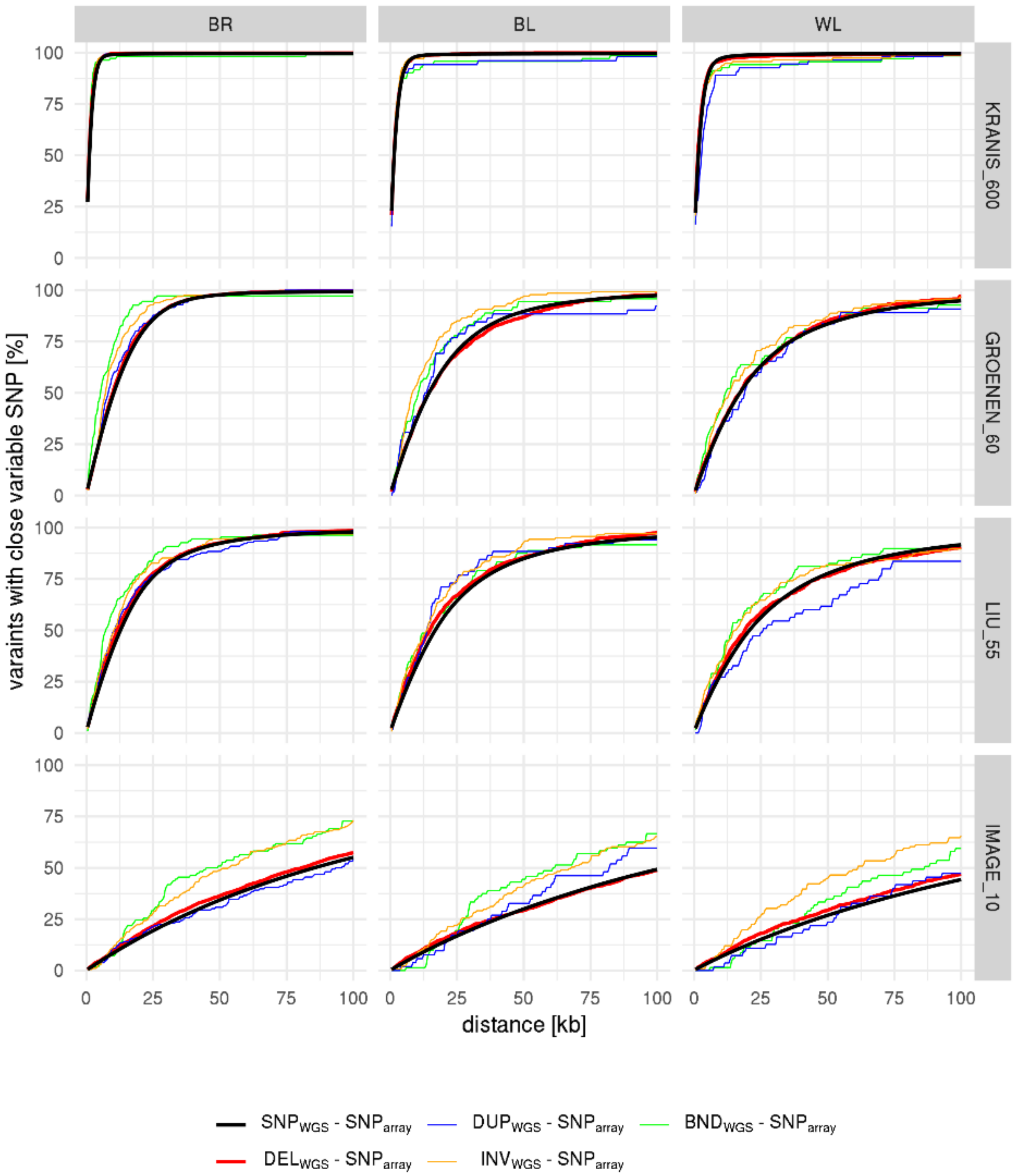

Figure S 16: Percentage of variants with at least one variable array SNP within a certain distance by distance and variant type for broiler (BR), brown layer (BL) and white layer (WL) chickens and four different genotyping arrays. KRANIS_600 = 600k Affymetrix array [3]; GROENEN_60 = 60k Illumina Bead Chip [4]; LIU_55 = 55k Affymetrix genotyping array [5]; IMAGE_10 = 10k Affymetrx genotyping array (IMAGE_001 multispecies array [6])

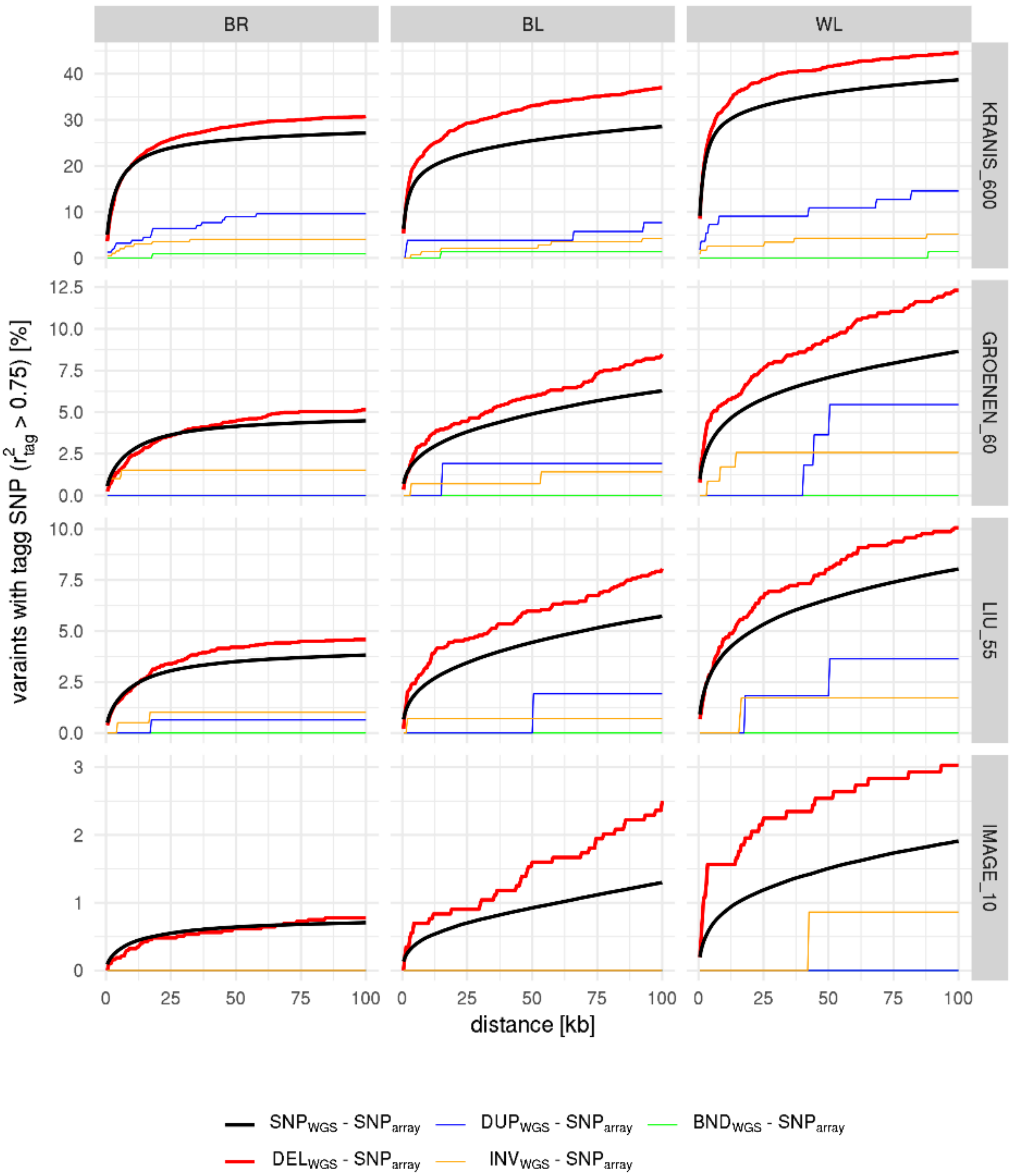

Figure S 17: Percentage of variants which are tagged by an array SNP within a certain distance ($r^2_{tag} > 0.75$) by variant type for broiler (BR), brown layer (BL) and white layer (WL) chickens and four different genotyping arrays. KRANIS_600 = 600k Affymetrix array [3]; GROENEN_60 = 60k Illumina Bead Chip [4]; LIU_55 = 55k Affymetrix genotyping array [5]; IMAGE_10 = 10k Affymetrix genotyping array (IMAGE_001 multispecies array r_S^2). Note that the y-axis is scaled to the according array.

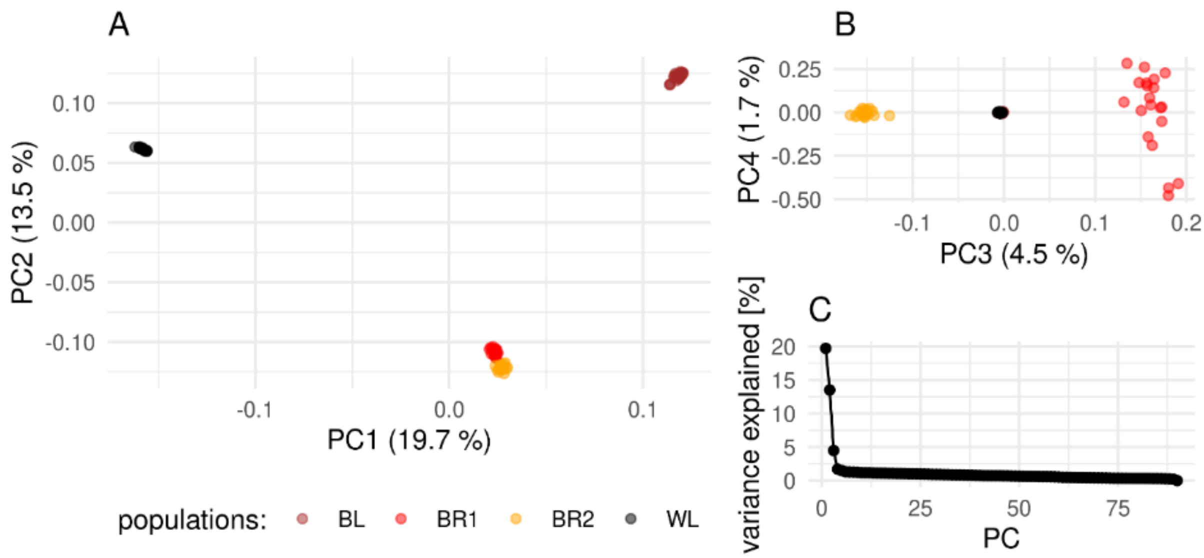

Figure S 18: Results of prime component analysis. First vs. second prime component (A), third vs. fourth prime component (B) and variance explained (C). The percentage of explained variance is denoted within brackets in the axis labels. BL – brown layer, BR1/2 – broiler, WL – white layer.

References

1. Pedersen BS, Quinlan AR. Duphold: scalable, depth-based annotation and curation of high-confidence structural variant calls. Gigascience. 2019;8:giz040. doi:10.1093/gigascience/giz040.
2. Graffelman J. Exploring Diallelic Genetic Markers: The HardyWeinberg Package. Journal of Statistical Software. 2015;64:1–23.
3. Kranis A, Gheyas AA, Boschiero C, Turner F, Le Yu, Smith S, et al. Development of a high density 600K SNP genotyping array for chicken. BMC genomics. 2013;14:59. doi:10.1186/1471-2164-14-59.
4. Groenen MAM, Megens H-J, Zare Y, Warren WC, Hillier LW, Crooijmans RPMA, et al. The development and characterization of a 60K SNP chip for chicken. BMC genomics. 2011;12:274. doi:10.1186/1471-2164-12-274.
5. Liu R, Xing S, Wang J, Zheng M, Cui H, Crooijmans RPMA, et al. A new chicken 55K SNP genotyping array. BMC genomics. 2019;20:410. doi:10.1186/s12864-019-5736-8.

6. Innovative Management of Animal Genetic Resources (IMAGE). DELIVERABLE D4.5: A standard multi-species chip for genomic assessment of collections. 2020. https://www.imageh2020.eu/deliverable/D4.5_resubmitted_final.pdf. Accessed 17 Aug 2021.
