# Supplementary material for: Assessment of linkage disequilibrium patterns between structural variants and single nucleotide polymorphisms in three commercial chicken populations
Source: BMC Genomics. 2022 Mar 9;23:193. doi: 10.1186/s12864-022-08418-7 (PMC8908679; doi:10.1186/s12864-022-08418-7)
Supplement: Supplementary file 3 — Additional file 3. Observer concordance of the visual filtering step. [file 12864_2022_8418_MOESM3_ESM.html]

Evaluate samplot Results


# Evaluate samplot Results

- Read Data
- Evaluate results
  - All SV
  - DEL
  - DUP
  - INV
  - BND
- Write results

# Read Data

```
library(data.table)
library(stringr)
library(ggplot2)
```

```
names <- c('Johannes','Paulina')
for(i in 1:length(names)){
  x <- data.table(name = list.files(path = paste0('D:/Promotion/LD_SV/samplot/prefiltered/bytype_',names[i],'/good')),
                  quality = 'good')
  x <- rbind(x,data.table(name = list.files(path = paste0('D:/Promotion/LD_SV/samplot/prefiltered/bytype_',names[i],'/bad')),
                          quality = 'bad'))
  x <- rbind(x,data.table(name = list.files(path = paste0('D:/Promotion/LD_SV/samplot/prefiltered/bytype_',names[i],'/review')),
                          quality = 'review'))
  x <- x[!is.na(name),]
  x[,name:=str_remove(name,'^[[:digit:]]+_')]
  x[,quality:=factor(quality,levels=c('good','bad','review'))]
  colnames(x)[2] <- names[i]
  if(i == 1){
    dat <- x
  }else{
    dat <- merge(dat,x)
  }
}
#str(dat)
dat[,type:=str_extract(name,'^[[:upper:]]+')]
dat
```

```
dat[Johannes == 'review',Johannes:='bad']
dat[Paulina == 'review',Paulina:='bad']
```

# Evaluate results

## All SV

```
table(dat[,.(Johannes,Paulina)])
```

```
##         Paulina
## Johannes good  bad review
##   good   4861   96      0
##   bad     108  157      0
##   review    0    0      0
```

```
kept <- table(dat[,.(Johannes,Paulina)])[1,1]/
  sum(table(dat[,.(Johannes,Paulina)]))
IOR <- sum(diag(table(dat[,.(Johannes,Paulina)])))/
  sum(table(dat[,.(Johannes,Paulina)]))
```

kept: 93.1 %

inter-observer-reliability: 96.1 %

## DEL

```
table(dat[type=='DEL',.(Johannes,Paulina)])
```

```
##         Paulina
## Johannes good  bad review
##   good   4301   77      0
##   bad      38   43      0
##   review    0    0      0
```

```
kept <- table(dat[type=='DEL',.(Johannes,Paulina)])[1,1]/
  sum(table(dat[type=='DEL',.(Johannes,Paulina)]))
IOR <- sum(diag(table(dat[type=='DEL',.(Johannes,Paulina)])))/
  sum(table(dat[type=='DEL',.(Johannes,Paulina)]))
```

kept: 96.5 %

inter-observer-reliability: 97.4 %

## DUP

```
table(dat[type=='DUP',.(Johannes,Paulina)])
```

```
##         Paulina
## Johannes good bad review
##   good    224   2      0
##   bad      18   8      0
##   review    0   0      0
```

```
kept <- table(dat[type=='DUP',.(Johannes,Paulina)])[1,1]/
  sum(table(dat[type=='DUP',.(Johannes,Paulina)]))
IOR <- sum(diag(table(dat[type=='DUP',.(Johannes,Paulina)])))/
  sum(table(dat[type=='DUP',.(Johannes,Paulina)]))
```

kept: 88.9 %

inter-observer-reliability: 92.1 %

## INV

```
table(dat[type=='INV',.(Johannes,Paulina)])
```

```
##         Paulina
## Johannes good bad review
##   good    219  16      0
##   bad      29  78      0
##   review    0   0      0
```

```
kept <- table(dat[type=='INV',.(Johannes,Paulina)])[1,1]/
  sum(table(dat[type=='INV',.(Johannes,Paulina)]))
IOR <- sum(diag(table(dat[type=='INV',.(Johannes,Paulina)])))/
  sum(table(dat[type=='INV',.(Johannes,Paulina)]))
```

kept: 64 %

inter-observer-reliability: 86.8 %

## BND

```
table(dat[type=='BND',.(Johannes,Paulina)])
```

```
##         Paulina
## Johannes good bad review
##   good    117   1      0
##   bad      23  28      0
##   review    0   0      0
```

```
kept <- table(dat[type=='BND',.(Johannes,Paulina)])[1,1]/
  sum(table(dat[type=='BND',.(Johannes,Paulina)]))
IOR <- sum(diag(table(dat[type=='BND',.(Johannes,Paulina)])))/
  sum(table(dat[type=='BND',.(Johannes,Paulina)]))
```

kept: 69.2 %

inter-observer-reliability: 85.8 %

# Write results

```
fwrite(dat,
       'D:/Promotion/LD_SV/samplot/prefiltered/res_Johannes_Paulina.csv',
       quote=FALSE)
```
